# Comparison of plant diversity-carbon storage relationships along altitudinal gradients in temperate forests and shrublands

**DOI:** 10.3389/fpls.2023.1120050

**Published:** 2023-08-11

**Authors:** Shuaizhi Lu, Dou Zhang, Le Wang, Lei Dong, Changcheng Liu, Dongjie Hou, Guoping Chen, Xianguo Qiao, Yuyouting Wang, Ke Guo

**Affiliations:** ^1^ State Key Laboratory of Vegetation and Environmental Change, Institute of Botany, Chinese Academy of Sciences, Beijing, China; ^2^ University of Chinese Academy of Sciences, Beijing, China; ^3^ Department of Environmental Science and Engineering, Fudan University, Shanghai, China; ^4^ Institute of Ecological Protection and Restoration, Chinese Academy of Forestry, Beijing, China; ^5^ Institute of Water Resources for Pastoral Area Ministry of Water Resources, Inner Mongolia, China; ^6^ Inner Mongolia Agricultural University, Hohhot, China; ^7^ Yunnan Climate Center, Kunming, China

**Keywords:** biomass carbon, forest and shrubland, structural diversity, species diversity, altitudinal gradients

## Abstract

Understanding the mechanisms underlying the relationship between biodiversity and ecosystem function (BEF) is critical for the implementation of productive and resilient ecosystem management. However, the differences in BEF relationships along altitudinal gradients between forests and shrublands are poorly understood, impeding the ability to manage terrestrial ecosystems and promote their carbon sinks. Using data from 37962 trees of 115 temperate forest and 134 shrubland plots of Taihang Mountains Priority Reserve, we analyzed the effects of species diversity, structural diversity, climate factors and soil moisture on carbon storage along altitudinal gradients in temperate forests and shrublands. We found that: (1) Structural diversity, rather than species diversity, mainly promoted carbon storage in forests. While species diversity had greater positive effect on carbon storage in shrublands. (2) Mean annual temperature (MAT) had a direct negative effect on forest carbon storage, and indirectly affected forest carbon storage by inhibiting structural diversity. In contrast, MAT promoted shrubland carbon storage directly and indirectly through the positive mediating effect of species diversity. (3) Increasing altitudinal gradients enhanced the structural diversity-carbon relationship in forests, but weakened the species diversity-carbon relationship in shrublands. Niche and architectural complementarity and different life strategies of forests and shrubs mainly explain these findings. These differential characteristics are critical for our comprehensive understanding of the BEF relationship and could help guide the differentiated management of forests and shrublands in reaction to environmental changes.

## 1 Introduction

Global environmental change, particularly global warming caused by increased carbon emissions from fossil fuels burning and direct land use change, is threatening an increasing number of species and their habitats, posing new challenges to ecosystem management (Friedlingstein et al., 2022). Global environmental change, such as temperature increase and precipitation change, not only directly affect biodiversity, but may also alter biodiversity and ecosystem function (BEF) relationship (Liang et al., 2016; Ratcliffe et al., 2017; Guo et al., 2019). Therefore, understanding the underlying mechanism of variations in BEF relationship under the context of global environmental change is critical for implementing productive and resilient ecosystem management and predicting the responses of plant physiological processes and functions to global environmental change.

Over the past few decades, most research has focused on understanding the role of niche complementarity, selection effects and their interrelationships in BEF through natural and experimental plant communities (Polley et al., 2003; Yachi and Loreau, 2007; Jing et al., 2015; Williams et al., 2017). The niche complementarity hypothesis, which states that improved ecosystem function is due to higher resource utilization through diverse species and functional traits within a community, generally explains a positive BFE relationship (Loreau and Hector, 2001; Yachi and Loreau, 2007). The selection hypothesis suggests that higher ecosystem function may be due to a higher probability of productive and high-functioning species in the community, which generally predicts a negative BEF relationship (Jing et al., 2015; Rodriguez-Hernandez et al., 2021). These mechanisms or ecological hypothesis that explain the BEF relationships are also applicable for the relationship between structural diversity and ecosystem function (Williams et al., 2017; Ali, 2019). So far, the relative importance of species diversity and structural diversity in promoting positive BEF relationships is the one of central debate (Poorter et al., 2015; Ali et al., 2016; Yuan et al., 2018). High species diversity leads to better utilization of resources in a community and reduces the competition among species based on the niche complementary mechanism, so it has a positive promoting effect on ecosystem function (Liang et al., 2016; Chen et al., 2018; Huang et al., 2018; Rahman et al., 2021). The positive relationship between species diversity and biomass carbon has been widely tested in several ecosystems with the urgent purposes of predicting the consequence of biodiversity loss and the potential for carbon sink (Tilman, 1999; Lasky et al., 2014; Ali and Yan, 2017; Yan et al., 2022).

However, recent studies have shown that structural diversity independently drives biomass carbon better than species diversity (Yuan et al., 2018; Ali et al., 2019a; Aponte et al., 2020; Wen et al., 2022). Structural diversity refers to the variation or heterogeneity of tree size (diameter, height, and/or crown) in a community (Ali et al., 2016; Danescu et al., 2016; Williams et al., 2017). The combination of individuals with different structures could allow a community to efficiently utilize resources (e.g., water, heat, light, soil nutrients), which is also attributed to niche differentiation and facilitation (Zhang et al., 2015; Glatthorn et al., 2017; Fotis et al., 2018). For example, variations in individual tree size can lead to improve canopy filling and spatial complementarity, thereby increasing light capture and utilization (Yachi and Loreau, 2007; Sapijanskas et al., 2014; Williams et al., 2017; Matsuo et al., 2021). Thus, structural diversity tends to increase biomass carbon through niche complementarity of individuals rather than species. Moreover, structural diversity is regarded as the mediated mechanism between species diversity and biomass carbon, as higher species diversity can drive diversification of stand structure (Zhang et al., 2015; Tan et al., 2017; Noulekoun et al., 2021).

Plant ecophysiology suggests that climatic factors, such as temperature and precipitation, have several distinct and convergent effects on ecosystem productivity and carbon storage (Chu et al., 2016; Corlett, 2016). Photosynthetic rate and respiration rate are the key physiological processes that determine the distribution of plant biomass, and are directly affected by temperature and water availability (Reich et al., 2018). In addition, climate factors can indirectly affect plant biomass by regulating the species composition and structural attributes of a community or ecosystem (Ratcliffe et al., 2017; Abbasi et al., 2022). Altitudinal gradients are the comprehensive reflection of local environmental conditions with different microclimates, water availability, and soil nutrients (Girardin et al., 2014; Noulekoun et al., 2021; Ullah et al., 2021; Wu et al., 2022), and thus could directly affect plant diversity by environmental filtering effect and indirectly affect carbon storage.

Although the plant diversity-carbon relationship has been widely reported in forests, the response mechanisms of ecosystem functions (e.g., biomass, carbon storage, or net primary productivity) to the variation of biotic and abiotic factors might vary in forests and shrublands (Guo et al., 2019; Yi et al., 2021) due to the differences in species dispersal and resource acquisition strategies (Biederman et al., 2018; Cao et al., 2020). Although only accounting for 10% of global terrestrial ecosystem (Pan et al., 2011; Yang et al., 2022), shrubland is still an irreplaceable component of terrestrial carbon sinks, especially in arid, cold, and disturbed lands where forests cannot grow (Biederman et al., 2018; Strassburg et al., 2020). Clarifying the BEF relationship between forests and shrublands can help us manage and improve terrestrial ecosystem carbon sinks.

Taihang Mountains Priority Reserve, one of China’s Biodiversity conservation priority areas released in 2015 (Wang et al., 2020), plays an irreplaceable role in maintaining ecosystem carbon sink function and protecting biodiversity in the Beijing-Tianjin-Hebei region. The vegetation in Taihang Mountains Priority Reserve are mainly secondary shrublands and semi-natural forests due to the historical deforestation (Wang et al., 2020). The secondary shrublands accounting for 47.55% of the total area of Taihang Mountains Priority Reserve are the main provider of regional ecosystem functions, and also the basis for the restoration and succession of temperate forest communities in the future. Faced with the demand of high-density population development in the Beijing-Tianjin-Hebei region and the task of eliminating historical carbon emissions, there is an urgent need for scientific ecosystem management to achieve the goal of carbon neutrality in China.

**Figure 1:**
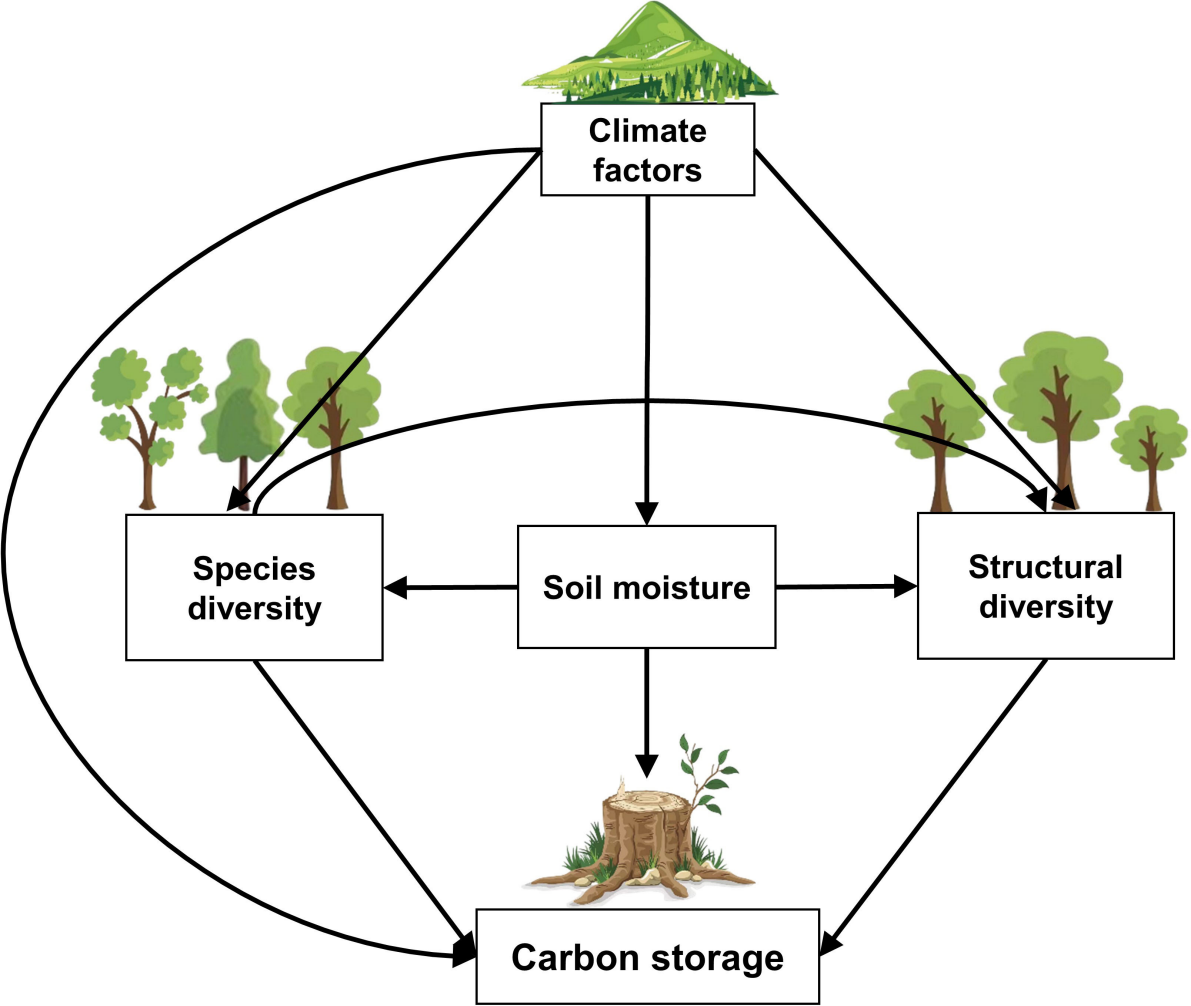
The hypothesized paths amongst carbon storage, species diversity, structural diversity, soil moisture, and climate factors in temperate forests and shrublands.

Therefore, using a composite structural equation model, with measured data of 37962 trees from 115 forest plots and 134 shrubland plots of Taihang Mountains Priority Reserve in North China, we examined the effects of species diversity, structural diversity, soil moisture and climate factors on carbon storage in temperate forests and shrublands (**Figure 1**; **Table 1**). The aim of our study is to answer three questions: (1) What are the differences in plant diversity-carbon relationships between temperate forests and shrublands? (2) How do environmental factors influence plant diversity and carbon storage? (3) How do altitudinal gradients modulate the plant diversity-carbon relationships in temperate forests and shrublands?

**Table 1:** Pathways and hypothesized mechanism based on piecewise structural equation modeling.

Number	Pathway	Hypothesized mechanism
1	Soil moisture → Carbon storage	Soil moisture affects plant carbon storage by regulating soil nutrient supply and uptake by plants (Green et al., 2019).
2	Species diversity → Carbon storage	Tree species diversity can affect plant carbon storage through several biological mechanisms, such as niche complementarity effect, selection effect (Huang et al., 2018).
3	Structural diversity → Carbon storage	High structural complexity of trees can enhance efficient use of resources for growth to promote plant carbon storage (Ali et al., 2019a; Aponte et al., 2020).
4	Climate factors → Carbon storage	Climate favorability affects plant growth and carbon storage by regulating respiration and water utilization (Chen et al., 2018).
5	Soil moisture → Species diversity	Soil moisture may affect plant diversity by regulating soil nutrient supply and uptake by plants, but the underlying mechanisms are unclear (Green et al., 2019).
6	Climate factors → Species diversity	Climate factors (i.e. temperature and precipitation) can cause alterations in species diversity with a momentous consequence for ecosystem carbon storage (Yan et al., 2022).
7	Soil moisture→ Structural diversity	Soil moisture may affect plant individual size by regulating soil nutrient supply and uptake by plants, but the underlying mechanisms are unclear (Green et al., 2019).
8	Species diversity→ Structural diversity	Species diversity plays a driving role in the complexity of stand structure, which indirectly increases carbon storage (Ali et al., 2016).
9	Climate factors → Structural diversity	Structural attributes and ecosystem properties and processes may vary along environmental gradients including temperature, rainfall and soil fertility (Poorter et al., 2015).
10	Climate factors → Soil moisture	Soil moisture is controlled by climatic forcing, particularly water balance (Green et al., 2019).

## 2 Materials and methods

### 2.1 Location and plot data

**Figure 2:**
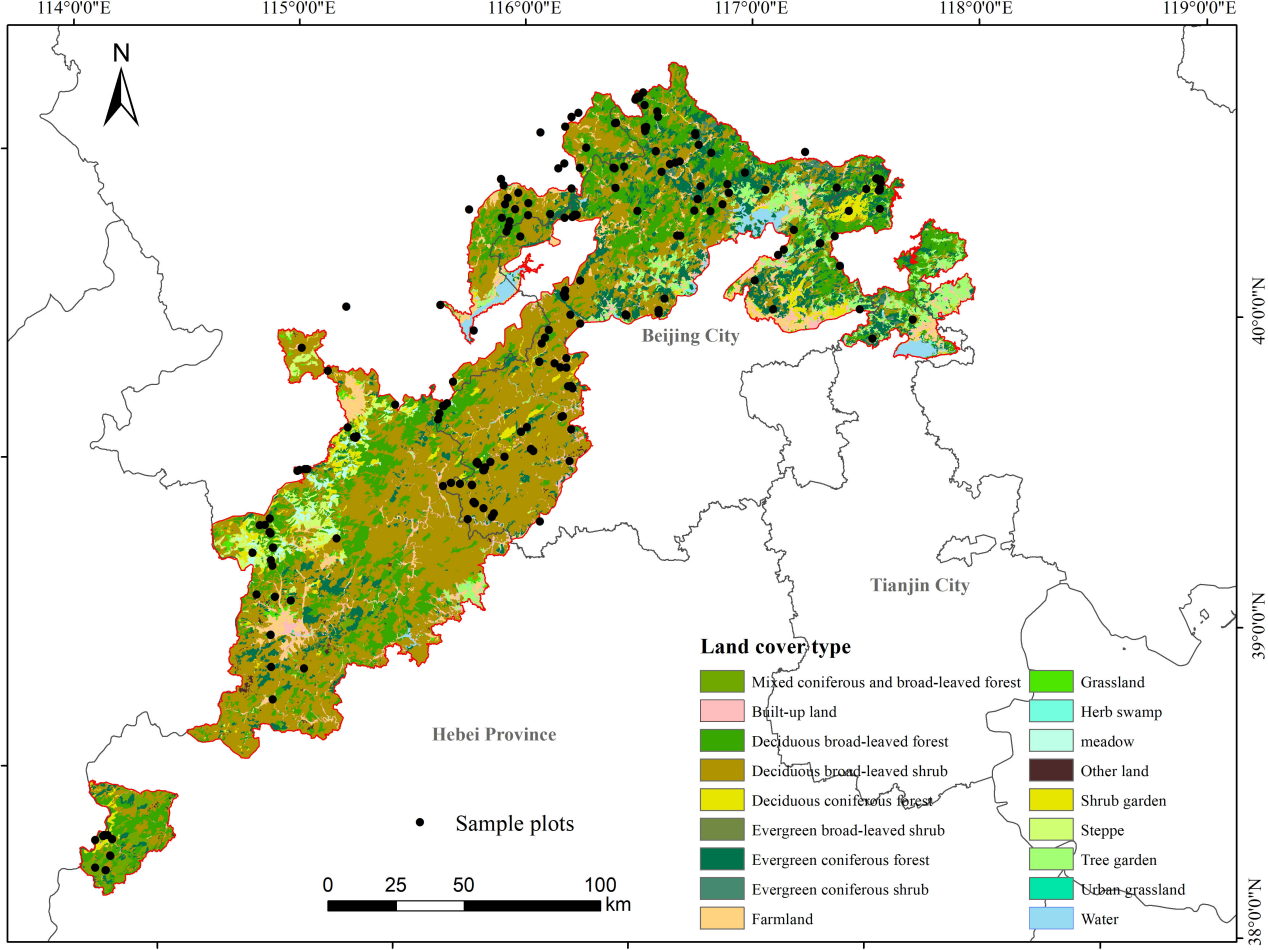
Location, sampling plots, and land use/cover of Taihang Mountains Priority Reserve.

Taihang Mountains Priority Reserve (38°33′13″-41°3′33″N, 113°41′32″-117°49′53″E) is one of the most important priority areas for biodiversity conservation in China. It covers an area of 2.1×10^4^ km^2^, spanning three administrative regions of Beijing, Tianjin City, and Hebei Province in China (**Figure 2**). The region belongs to a mountainous climate in the transition zone from semi-humid to semi-arid. Altitude in this region ranges from 93 to 2882 m. Annual average temperature ranged from 5 to 11 °C, and the annual precipitation ranged from 400 to 800 mm.

The major vegetation types in Taihang Mountains Priority Reserve are temperate deciduous broad-leaved shrubland temperate deciduous broad-leaved forest, and evergreen coniferous forest, accounting for 47.55%, 20.15% and 11.26% of the total vegetation area respectively (Wang et al., 2020). The main dominant species of shrubs include *Vitex negundo* var. *heterophylla*, *Ziziphus jujuba* var. *spinose*, *Armeniaca sibirica*, *Amygdalus davidiana*, and *Ostryopsis davidiana*. The main tree species include *Quercus mongolica*, *Betula platyphylla*, *Populus davidiana*, *Pinus tabuliformis*, and *Platycladus orientalis*. This region has a relatively well-preserved warm temperate forest of North China with rich biodiversity, which is of great conservation value. Meantime, faced with the increasingly intense human disturbance, it is urgent to implement the scientific forest ecosystem management and protection to enhance ecosystem functioning.

We conducted the field investigation throughout the study area in the summer (August to October) of 2019 and 2020. A total of 249 plots were investigated along the altitude range of 93 m ~ 2391 m, including 134 shrubland plots and 115 forest plots. The shrubland plots were all deciduous broad-leaved shrubland plots, and the forest plots included 82 plots of the deciduous broad-leaved forest, 22 plots of evergreen coniferous forest, and 11 plots of deciduous coniferous forest (**Figure 2**). In our field investigation, we divided trees and shrubs mainly according to plant species and a widely used criteria for forest plot investigation where trees below 3cm in DBH are classified as shrubs (Fang et al., 2009). The area of the forest plot was 20 m × 20 m, and the observation records included tree layer and shrub layer. For tree layer, all tree species, DBH, height and crown width for each individual with DBH≥3 cm were measured and recorded. For shrub layer, four squares with an area of 10 m×10 m were selected for investigation, and all shrub species, basal diameter, height and crown width were measured and recorded. Finally, our study recorded a total of 37962 trees of 198 species belonging to 94 genera and 45 families.

### 2.2 Carbon storage estimation

We calculated both the aboveground and belowground biomass of each tree and shrub using allometric growth equations (Eqs.1) collected for different species and tree components including stems, branches, leaves, and roots. For the plant species without a specific equation, we used a general equation for the genus, family, or coenotype to which a plant species belongs. All allometric growth equations used in this study were in **Table S1**. Woody plant biomass was multiplied by organic carbon content to obtain carbon storage. In order to improve the accuracy, we used different organic carbon content depending on tree species and tree components based on the previous measurements in the same region (Ma et al., 2002) (**Table S1**). We then summed the living wood carbon storage of all individual trees for each plot. Finally, carbon storage density (Mg/ha) of each plot was calculated.


(1) $$W\;=a{\left(D^{2}H\right)}^{b}$$

Where, *W* is biomass of individual; *D* is the diameter at breast height; *H* is the tree height; *a*, *b* are the model coefficients.

### 2.3 Plant diversity and environmental variables

For species diversity, we calculated the species richness (SR) (i.e., the number of woody plant species within a plot), Shannon-Wiener index (SW) (Eq.(2)), Simpson index (SI) (Eq.(3)), and Shannon evenness (SE) (Eq.(4)), which are the most commonly used indices for assessing species diversity (Ali et al., 2019a). Shannon-Wiener index assumes that heterogeneity depends on species richness and evenness within a community, which is similar to Simpson index, but Shannon-Wiener index is more sensitive to rare species. Shannon evenness is an indicator that measures the relative abundance of different species that make up the species richness within a community.


(2) $$H_{S}\;=\;-\sum_{i=1}^{S}P_{i}\times ln(P_{i})$$

(3) $$D_{S}=1-\sum_{i=1}^{S}P_{i}^{2}$$

(4) $$J_{S}=\frac{H_{S}}{ln(S)}$$

Where *H_s_* is Shannon-Wiener index; *D_s_* is Simpson index; *J_s_* is Shannonevenness; *P_i_* is the proportion of basal areas of species *i*; *S* is the number of woody plant species within a plot.

For structural diversity, we quantified diameter at breast height (DBH) diversity, height diversity, and crown width diversity by calculating the coefficient of variation of DBH (CV*_d_*), height (CV*_h_*), and crown width (CV*_c_*) in each plot. For shrubs, we calculated the structural diversity index using basal diameter instead of DBH (Yi et al., 2021). The formulas are as follows:


(5) $$CV_{d}\;=\;100*\frac{SD_{d}}{Mean_{d}}$$

(6) $$CV_{h}\;=\;100*\frac{SD_{h}}{Mean_{h}}$$

(7) $$CV_{c}\;=\;100*\frac{SD_{c}}{Mean_{c}}$$

where *SD_d_*, *SD_h_*, and *SD_c_* are the standard deviations of DBH, height and crown width in each plot respectively. *Mean_d_*, *Mean_h_*, and *Mean_c_* are the mean values of DBH, height and crown width in each plot respectively. For shrubs, *SD_d_* and *Mean_d_* refer to the standard deviation and mean value of basal diameter respectively. A higher CV reflects a higher-level structural diversity, which is a more complex structure. The calculation for all diversity indices was performed with vegan package of R4.2.1.

In addition, we calculated the mean annual temperature (MAT) and mean annual precipitation (MAP) of the study area using data from 177 meteorological stations in the Beijing-Tianjin-Hebei region. The meteorological data were obtained from the China Meteorological Science Data Sharing Service Network (http://cdc.cma.gov.cn). The soil moisture data was obtained from a 1 km daily soil moisture dataset over China using *in situ* measurement (Li et al., 2022), which was provided by National Tibetan Plateau Data Center (http://data.tpdc.ac.cn). To explore how altitudinal gradients influence the relationship between plant diversity and carbon storage, we divided forest and shrubland plots into high, middle and low altitudes according to the widely used classification criteria of hilly (below 500 m) and mountainous (above 500 m) in China and the vertical distribution characteristics of tree species in the study area (Wang et al., 2020), using the altitude measured at the site. Finally, high, middle and low altitudes in our study correspond to >1000m, 500~1000m, and 0~500m respectively.

### 2.4 Piecewise structural equation model and statistical analysis

In this study, we established a composite piecewise structural equation model (pSEM) (Tian et al., 2021; Liu et al., 2022), which could incorporate multiple independent variables in each path, to analyze the effects of composite structural diversity, species diversity as well as environmental factors on woody plant carbon storage. Before performing the composite pSEM, we examined the bivariate relationships among all tested variables calculated in Sections 2.2 and 2.3 by Pearson correlation analysis (**Figures S1**, **S2**). And if the correlation between a factor and carbon storage is not statistically significant in both forest and shrubland plots, the factor will be eliminated from pSEM, such as CV*_c_*, Shannon evenness (SE), and mean annual precipitation (MAP). Therefore, the indices of CV*_d_*, CV*_h_*, species richness (SR) index, Shannon-Wiener (SW) index, Simpson (SI) index, mean annual temperature (MAT) and soil moisture (SM) were finally selected to be included in pSEM analysis. Then, the composite structural diversity variable was calculated as follows in R 4.2.1. First, we obtained the corresponding regression standard coefficients by conducting linear regression analysis between carbon storage and CV*_d_*, CV*_h_*. Then, we calculated the composite structural diversity variable by multiplying and summing the regression standard coefficients of CV*_d_*, CV*_h_* with corresponding original variables. Similarly, we calculated composite species diversity variable by integrating SR, SW and SI indices.

In order to satisfy the linearity and normality of the data, and to compare the relative influence of multiple predictors on the response variables, all variables were natural log-transformed and Z-score standardized before statistical analysis in IBM SPSS Statistics 20. The model-fit to data of the composite pSEM was evaluated by Fisher’s C statistics with P-value. Fisher’s C with P > 0.05 indicates a goodness-of-fit. The composite pSEM and all statistical analyses were conducted mainly using *ggcorplot, nmle*, *lme4* and *piecewiseSEM* packages in R. 4.2.1.

## 3 Results

### 3.1 Bivariate relationships of all hypothesized paths in pSEM

**Figure 3:**
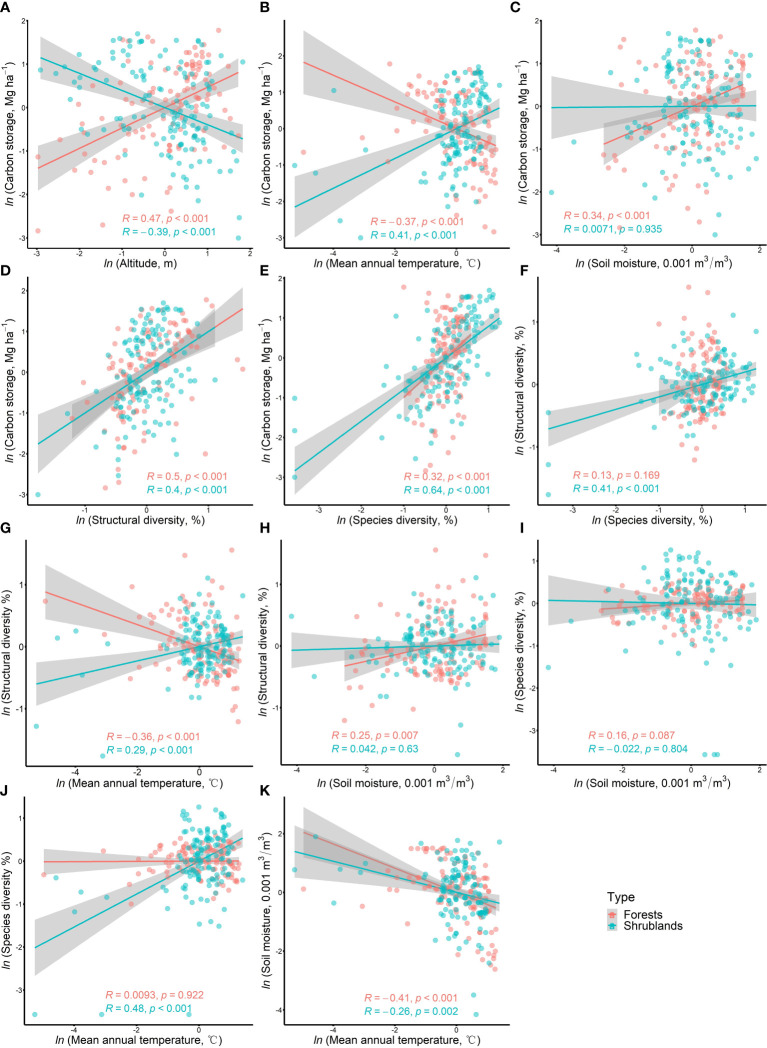
Simple bivariate relationships among all hypothetical paths in pSEMin forests and shrublands. The solid lines are fitted regression line given with Pearson correlation coefficient (R) and P-value. The gray shading represents their 95% confidence band.

The bivariate relationships for all hypothesized paths in forests and shrublands were shown in **Figure 3**. The simple bivariate relationship between carbon storage and plant diversity (structural and species diversity) was consistent in forests and shrublands, showing positive correlation (**Figures 3D, E**). However, the simple bivariate relationship between carbon storage and altitude as well as mean annual temperature showed the opposite trend in forests and shrublands (**Figures 3A, B**). In forests, carbon storage significantly (P< 0.05) increased with increasing altitude, soil moisture, structural diversity and species diversity, but significantly decreased with increasing mean annual temperature (**Figures 3A–E**). In shrublands, carbon storage significantly increased with increasing mean annual temperature, structural diversity and species diversity (**Figures 3B, D, E**), but decreased with increasing altitude (**Figure 3A**). Besides, the simple bivariate relationship between structural diversity and mean annual temperature also showed the opposite trend in forests and shrublands (**Figure 3G**). Structural diversity significantly decreased with increasing mean annual temperature in forests, but increased in shrublands. Species diversity significantly decreased with increasing mean annual temperature in both forests and shrublands. In general, from a simple bivariate relationship, carbon storage in forests and shrublands had consistent responses to plant diversity (structural and species diversity), but opposite responses to altitude and mean annual temperature.

### 3.2 Direct and indirect effects on forest and shrubland carbon storage

**Figure 4:**
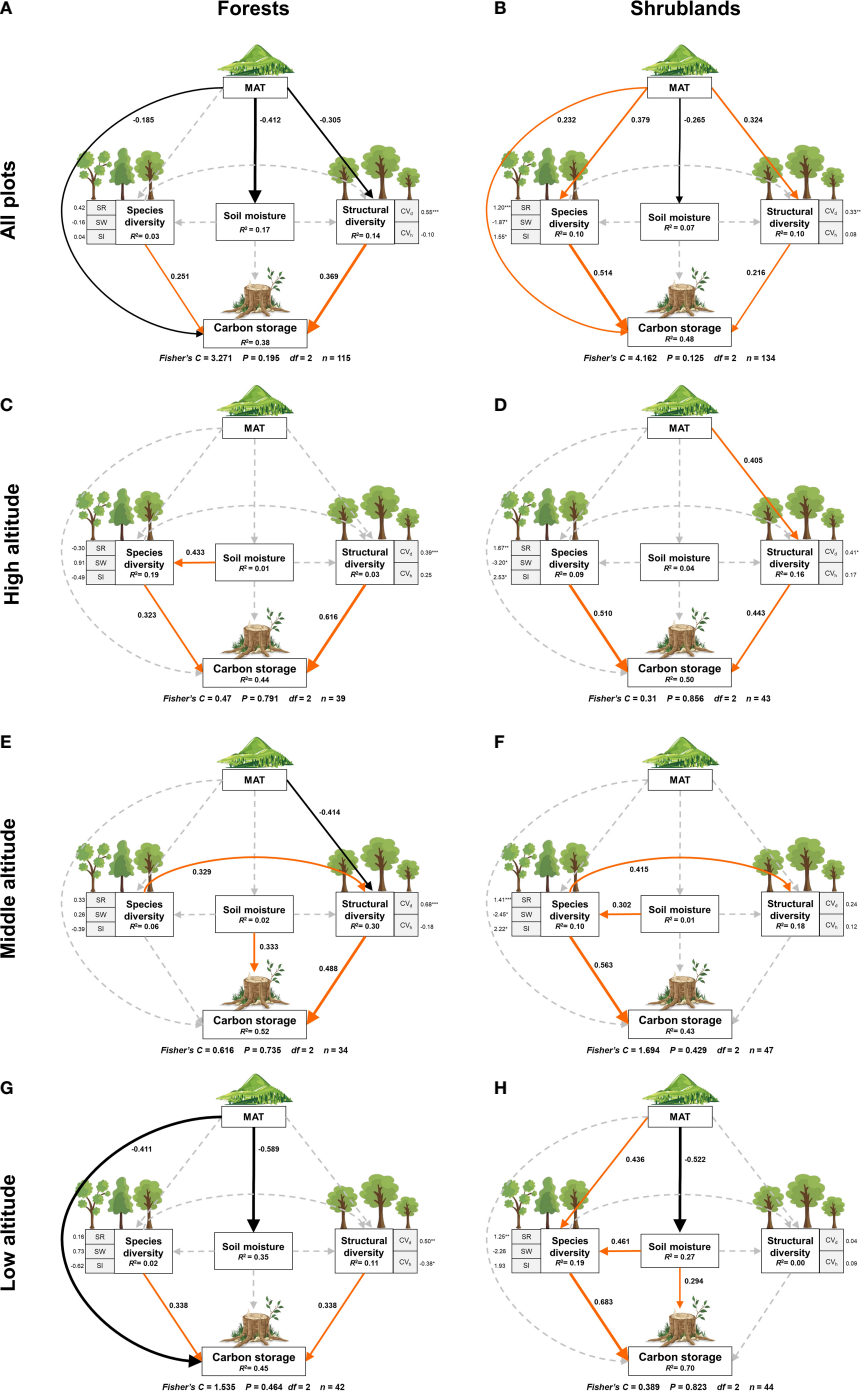
The composite pSEM linking species diversity, structural diversity, mean annual temperature and soil moisture to carbon storage in temperate forests and shrublands. Solid red and black arrows represent significant positive and negative paths (P< 0.05) respectively, while dashed gray arrows show non-significant paths (P > 0.05). The numbers on the path are the standard path coefficients. Numbers adjacent to measured variables are their coefficients with composite variables. R^2^ represents the proportion of variance of each response variable explained by the predictors. MAT, mean annual temperature; CV*_d_*, coefficient of variation in diameter at breast height; CV*_c_*, coefficient of variation in tree height; SR, species richness index; SW, Shannon-Wiener index; SI, Simpson index.

The composite pSEM showed that mean annual temperature, soil moisture, structural diversity and species diversity together explained 38% and 48% of the variation in forest and shrubland carbon storage, respectively (**Figures 4A, B**). The model also explained 17%, 14% and 3% of the variation in soil moisture, structural diversity and species diversity in forests, respectively, and 10%, 10% and 7% of the variation in species diversity, structural diversity and soil moisture in shrublands, respectively.

In forests, structural diversity had a strongest direct positive effect (0.369, P< 0.001; **Figure 4A**; **Table S2**) on carbon storage, followed by species diversity (0.251, P< 0.01), while mean annual temperature had a direct negative effect (-0.185, P< 0.05) on carbon storage. The results suggested that structural diversity, not species diversity, determined the distribution of forest carbon storage in this area. In addition to a direct effect, mean annual temperature also had an indirect negative effect on carbon storage *via* a negative effect on structural diversity (-0.113 = -0.305*0.369). In other words, with the decrease of mean annual temperature, structural diversity increased, leading to an indirect increase of carbon storage. It showed that structural diversity mediated the negative indirect effect of mean annual temperature on carbon storage in forests.

In shrublands, species diversity had a strongest direct positive effect (0.514, P< 0.001; **Figure 4B**; **Table S6**) on carbon storage, followed by mean annual temperature (0.232, P< 0.01) and structural diversity (0.216, P< 0.01). Unlike forests, the results suggested that species diversity determined the distribution of carbon storage in shrublands. In addition to a direct effect, mean annual temperature also had an indirect positive effect on shrubland carbon storage *via* a positive impact on species diversity (0.195 = 0.379*0.514) and structural diversity (0.070 = 0.324*0.216), which meant that with the increase of mean annual temperature, species diversity and structural diversity increased, leading to the increase of carbon storage. Species diversity played a stronger intermediary role on the indirect effect of mean annual temperature on carbon storage in shrublands. The relationship between soil moisture and carbon storage was not significant (P > 0.05) in both forests and shrublands.

**Figure 5:**
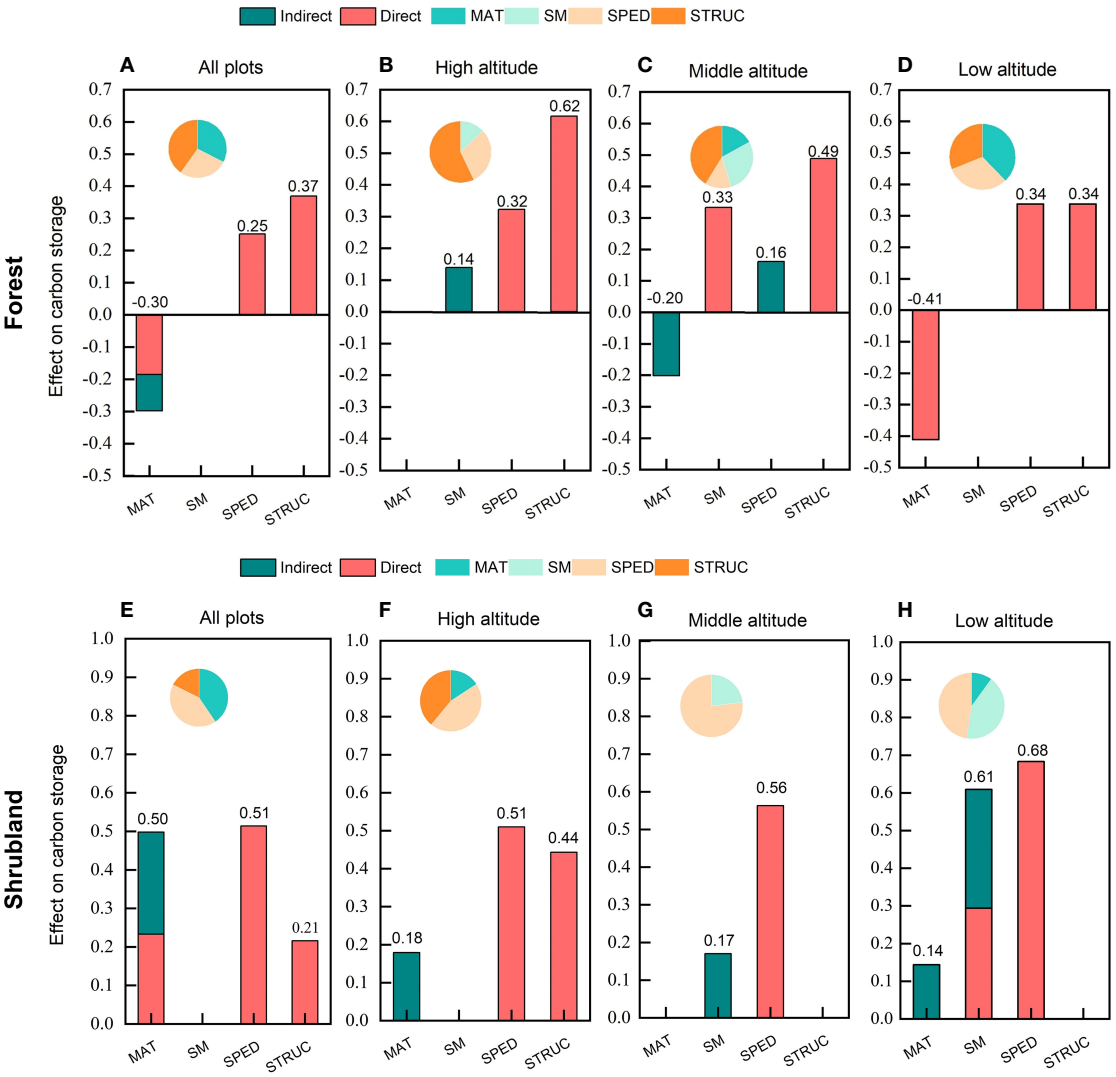
Direct, indirect and total standardized effects of mean annual temperature, soil moisture, species diversity, structural diversity on carbon storage in temperate forests and shrublands. The pie chart shows the proportion of standard effect of each factor. MAT, mean annual temperature; SM, soil moisture; SPED, species diversity; STRUC, structural diversity.

By visualizing the direct, indirect and total effects of all predictors on carbon storage (**Figure 5**), we found that structural diversity (0.37) was the main driver of carbon storage in forests with the strongest total effect, followed by mean annual temperature (-0.30), and species diversity (0.25) (**Figure 5A**). Whereas, species diversity (0.51) was the main driver of carbon storage in shrublands with a more than half proportion of relative effect, followed by mean annual temperature (0.50) and structural diversity (0.21) (**Figure 5D**). It worth noting that mean annual temperature had the indirect effect on carbon storage in both forests and shrublands. See all details of direct, indirect and total effects of the composite pSEM for forest and shrublands in **Table S2** and **S6** respectively.

### 3.3 Plant diversity-carbon relationships along the altitudinal gradients

We further analyzed the plant diversity-carbon relationship at different altitudinal gradients. The results of the composite pSEMs in forests and shrublands at altitudinal gradients were in **Tables S3–S5, 7–9**. In forests, the pSEMs of high, middle and low altitudes explained 44%, 52% and 45% of the carbon storage respectively (**Figures 4C, E, G**). We found that the direct positive effect of structural diversity on carbon storage enhanced significantly with the altitudinal gradients (local environmental gradients) in forests. The direct positive effect of structural diversity on carbon storage at high altitude (0.616, P< 0.001; **Table S3**) was almost twice that at low altitude (0.338, P< 0.05; **Table S5**). Altitudinal gradients also altered the relationship between species diversity and carbon storage. Specifically, species diversity had a direct positive effect on carbon storage at high (0.323, P< 0.05; **Table S3**) and low altitude (0.338, P< 0.01; **Table S5**) and had an indirect positive effect through a positive influence on structural diversity at middle altitude (0.161 = 0.329*0.489; **Table S4**). It is worth noting that the direct effect of species diversity on carbon storage became equal to that of structural diversity at low altitude, although structural diversity played a dominant role in forest carbon storage throughout the study area (**Figure 4A**). Besides, the effects of mean annual temperature on carbon storage also changed with altitudinal gradients. Mean annual temperature had a direct negative effect on carbon storage at low altitude (-0.411, P< 0.05; **Table S5**) and an indirect negative effect through a negative impact on structural diversity at middle altitude (-0.201 = -0.414*0.489), but was not significant at high altitude. Soil moisture only had a significant direct positive effect on carbon storage at middle altitude (0.333, P< 0.05).

In shrublands, the pSEMs of high, middle and low altitudes explained 50%, 43% and 70% of the carbon storage respectively (**Figures 4D, F, H**). In contrast to the forests, the direct positive effect of species diversity on carbon storage weakened significantly with the altitudinal gradient (local environmental gradient) in shrublands. The direct positive effect of species diversity on carbon storage at high altitude (0.510, P< 0.001; **Table S7**) was much less than that at low altitude (0.683, P< 0.001; **Table S9**). And at high altitude, the positive effect of structural diversity on shrubland carbon storage became significant (0.443, P< 0.005; **Table S7**). Moreover, altitudinal gradients changed the indirect effect of mean annual temperature on carbon storage. Mean annual temperature had an indirect negative effect on carbon storage mediated by soil moisture (-0.154 = -0.522 *0.294), but indirect positive effect mediated by species diversity (0.298 = 0.683*0.436) at low altitude but had indirect positive effect mediated by structural diversity (0.179 = 0.405*0.443) at high altitude. It worth noting that the direct positive effect of soil moisture on carbon storage became significant at low altitude (0.294, P< 0.05; **Table S9**).

Altitudinal gradients also changed the relative effects (the proportion of standard path coefficients) of predictors on carbon storage (**Figure 5**). The relative effect ratio of structural diversity on forest carbon storage enhanced steadily with the increasing altitude. Specifically, in terms of total effect, structural diversity was the main driver of forest carbon storage at high and middle altitudes (0.62 and 0.49) (**Figures 5B, C**), but at low altitude, mean annual temperature became the main driver of carbon storage with a negative effect of -0.41 and the role of species diversity was starting to loom large (**Figure 5D**). In shrublands, the relative effect of species diversity weakened with increasing altitude, but species diversity was still the most important driver of carbon storage at different altitudes (**Figures 5F–H**). The results indicated that the high altitude (accompanied by lower temperature and higher soil moisture; **Figures S1** and **S2**) enhanced the dominant role of structural diversity on forest carbon storage, but weaken the dominant role of species diversity on shrubland carbon storage.

## 4 Discussion

To the best of our knowledge, this is the first study to systematically compare forest and shrubland woody plant carbon storage in relation to their biotic and abiotic drivers (structural diversity, species diversity, mean annual temperature and soil moisture). Based on 249 temperate forest and shrub plots of the uniform vertical gradient distribution, we found that: (1) both structural diversity and species diversity positively drove woody plant carbon storage, but their dominant roles were different in forest and shrubland ecosystems; (2) Climate factors (i.e., temperature) not only directly affect carbon storage, but also indirectly affect carbon storage by affecting species diversity and structural diversity; (3) In addition, altitudinal gradients altered the plant diversity-carbon relationship in forest and shrubland ecosystems.

### 4.1 Plant diversity-carbon relationship and its differences in forest and shrubland

First, our pSEM of temperate forest plots showed that structural diversity, rather than species diversity, mainly determined forest carbon storage. This is consistent with recent studies of different ecosystems including tropical forests (Poorter et al., 2015; Ali et al., 2019a; Wen et al., 2022), subtropical forests (Ali et al., 2016), temperate forests (Yuan et al., 2018; Aponte et al., 2020) and dry Afromontane forests (Tetemke et al., 2021) (**Table S10**). Structural diversity is increasingly recognized as an important determinant of forest carbon storage. Several important processes and mechanisms have been shown to increase light capture of complex tree structure, i.e., spatial complementarity effect, and thus carbon storage: (a) architectural niche differentiation, and (b) crown plasticity (Danescu et al., 2016; Schnabel et al., 2019). Tree size variation lead to leaf stratification and multilayer canopy, which affects ecological processes such as photosynthesis and respiration as well as forest carbon storage (Poorter et al., 2015). Therefore, high structural diversity will enhance spatial complementarity effects through efficient light capture and utilization, while low structural complexity may weaken niche complementarity effects (Ali et al., 2016; Williams et al., 2017).

In our study, the composite structural diversity constructed by diameter and height diversity index (CV*_d_* and CV*_h_*) can reflect the degree of architectural niche differentiation and crown plasticity, thus promoting niche complementary effect (Danescu et al., 2016; Kazempour Larsary et al., 2021). And the main tree species analyzed in the study have significant individual size differences, occupy different canopy locations, and have canopy spatial complementary properties (Williams et al., 2017), thus improving light capture and light use efficiency (Atkins et al., 2018). The higher positive Pearson correlation coefficients between diameter, height diversity (CV*_d_* and CV*_h_*) and forest carbon storage compared to species diversity indices (**Figure S1**), supporting a more important role of structural diversity in forest carbon storage. In addition, structural diversity may be related to species/functional diversity, as increased species/functional diversity may lead to occupying more spatial niches (Zhang et al., 2015). However, no significant positive correlation between species diversity and structural diversity was detected in our pSEMs except in forests at middle altitude, because single and species-poor stands can still have structural diversity through vertical and horizontal differentiation of a limited number of species (Tetemke et al., 2021).

Similarly, consistent with the most previous studies, our pSEM results also support a positive species diversity-carbon relationship (Zhang et al., 2016; Chen et al., 2018; Huang et al., 2018; Rahman et al., 2021). Systematic experimental and theoretical studies have shown that species diversity can positively affect plant carbon storage through two main non-exclusive mechanisms: niche complementarity effect (the complementarity of different species in terms of function and resource utilization) (Yachi and Loreau, 2007; Zhang et al., 2016; Mensah et al., 2018), and selection effect (strong productivity advantage of species with high levels of function) (Loreau and Hector, 2001). The species diversity indices used in our study, including species richness, Shannon-Wiener index and Simpson index, can reflect niche complementarity effect to some extent, that is, the diversity of species and their niche enhances ecosystem function through the effective resource utilization by constituent species within a community (Tilman, 1999; Ali et al., 2019a). Although most of our results supported a positive diversity-carbon relationship, insignificant results still existed, such as the species diversity-carbon relationship in forests at middle and high altitudes (**Figures 4C, E**). These controversial results suggest that the effects of species diversity on plant carbon storage may vary depending on resource utilization conditions in different environments (Ratcliffe et al., 2017; Jucker et al., 2018; Ali et al., 2019a).

Unlike forests, our pSEMs showed that species diversity had a greater effect on carbon storage than structural diversity in shrubland ecosystems (**Figure 4B**). This is because the individual size variation of shrubs is significantly lower than that of forests, and the variation degree of structural diversity is lower. In this case, high species richness and functional trait diversity bring higher carbon storage under the niche complementarity effect (Poorter et al., 2015; Yi et al., 2021). Theoretically, species diversity leads to aboveground and underground niche differentiation (complementary of multiple resources), while the effects of structural diversity on forest carbon storage mainly come from aboveground spatial niche differentiation (light capture and utilization), which is a subset of the impacts of species diversity (Schnabel et al., 2019). Thus, species diversity dominated the positive diversity-carbon stock relationship in shrubland ecosystems with relatively simple structures.

### 4.2 Altitudinal gradients regulate plant diversity-carbon relationship

Altitudinal gradients may lead to micro-environmental heterogeneity in resource availability (light, water, and soil nutrients) (Jucker et al., 2018; Ali, 2023). A higher altitudinal gradient receives lower temperature and higher soil moisture content (Liu Z. et al., 2022). The altitude span of our study area exceeds 2000m, and the vertical temperature span can reach 6 degrees. Therefore, with the increase of altitude, temperature would significantly decrease, soil moisture increases significantly and thus affect species distribution as well as their diversity characteristics, and carbon accumulation (Girardin et al., 2014; Ratcliffe et al., 2017; Ali et al., 2019b). Forest and shrubland have significantly different responses to environmental factors such as temperature and soil moisture. From our previous study on vegetation diversity and mapping in Taihang Mountains Priority Reserve (Wang et al., 2020), temperate shrubland is more tolerant to drought and heat than forest (Werner and Homeier, 2015; Xia et al., 2016; Jucker et al., 2018). To be specific, at low altitude, broad-leaved forests do not grow well because of the limited water availability caused by higher temperature and evaporation. As a result, shrubs such as *Armeniaca sibirica* and *Vitex negundo L.* var. *heterophylla*, and drought-tolerant forests, such as *Platycladus orientalis*, are mainly distributed at low altitude. Then at middle altitude, broad-leaved forests begin to appear on the shady slope due to decreased temperature and increased humidity, and the drought-tolerant coniferous forest, *Quercus mongolica* and *Pinus tabulaeformis* dominate on the sunny slope. As the altitude continued to increase (at high altitude), temperature rather than water availability became the main limiting factor, the vegetation is dominated by *Betula platyphylla* and hardy coniferous. These analyses explain the differential responses of forest and shrubland to altitude or temperature condition changes.

Our pSEMs suggested that high altitude prominently strengthened the positive relationship of structural diversity-carbon in forest ecosystem. We speculated that forests at high altitudes might have a higher structural diversity, closed to natural forests due to relative slight disturbance from anthropogenic activities. Pearson correlation results (**Figure S1**) also showed that structural diversity increased significantly with altitude, supporting our speculation. Specifically, the dominant species at high altitudes are *Betula platyphylla*, *Picea wilsonii* and *Picea meyeri*. *Betula platyphylla* is often inlaid and mixed with other *Betula*, *Quercus mongolica*, *Populus davidiana*, *Tilia tuan*, etc., or form *theropencedrymion* with *Larix principis-rupprechtii*, resulting in a complex forest composition and structure (Wang et al., 2020). A higher structural diversity leads to more efficient light capture, carbon capture and storage through canopy filling according to the niche complementary hypothesis (Glatthorn et al., 2017; Aponte et al., 2020; Noulekoun et al., 2021). Our Pearson correlation analysis results showed that there forest carbon storage and soil moisture significantly increased with altitude (**Figure S1**), which also reflected that the altitude in this region had not reached the extreme low temperature limit on forest growth. The more adequate moisture condition (high humidity and soil moisture) at high altitude made coniferous forests with high carbon storage widely distributed. These analyses explain the role of altitude in regulating the forest structural diversity-carbon relationship through environmental factors (temperature and soil moisture).

Secondly, our pSEMs also found that high altitude weakened the positive relationship of carbon-species diversity in shrublands. Combined with Pearson’s correlation results that species diversity (SR, SW, SI) in shrubland decreased significantly with increasing altitude (**Figure S2**), we speculated that this might be attributable to relatively poor shrub species and less efficient resource utilization due to environmental filtering at high altitude. Previous studies have shown that the effect of species diversity on carbon storage tend to be weaker in species poor condition with limited resource utilization (Van Der Sande et al., 2018; Aponte et al., 2020). Theory suggests that overyielding in mixed forests is expected to increase only if interspecies interactions increase water use efficiency as water conditions are limited (Forrester, 2014; Forrester and Bauhus, 2016). Our results support this theory, that is, species diversity has the strongest complementary effect on shrubland carbon storage in arid low-altitude areas characterized by higher temperature and lower soil moisture (**Figures 5H** and **S2**), while the positive effect of species diversity was significantly weakened at high altitude with relatively humid and low temperature (**Figures 5D** and **S2**).

## 5 Conclusion

The results of this study suggest that both structural diversity and species diversity can positively contribute to temperate forest and shrubland carbon storage, but structural diversity dominates forest carbon storage and species diversity dominates shrubland carbon storage. High altitude enhanced the effect of structural diversity on forest carbon storage, but weakened the effect of species diversity on shrubland carbon storage. These differential characteristics should be taken into account in future sustainable forest management decisions. For forests, choosing a combination of different tree sizes according to altitude to maintain a complex stand structure will contribute to higher productivity. For shrublands, the selection of suitable species combinations based on altitude to maintain a high niche complementary effect will help achieve the management objectives of biodiversity conservation and increased carbon sink. In addition, abiotic factors played an important regulatory role in the process of directly or indirectly affecting carbon storage, and differentiated planting patterns along the altitudinal gradient should be adopted to maintain ecosystem functions. More environmental determinants (such as climatic water and soil water availability), plant functional traits and nonlinear relationships should be fully considered in the future, which is important for maintaining high productivity and carbon storage, especially under changing environmental conditions in the future.

## Data availability statement

The original contributions presented in the study are included in the article/**Supplementary Material**. Further inquiries can be directed to the corresponding author.

## Author contributions

SL, DZ, and KG contributed to conception and design of the study. LW, LD, and CL organized the database. SL and DZ performed the statistical analysis and wrote the first draft of the manuscript. DH, GC, XQ, and YW wrote sections of the manuscript. All authors contributed to manuscript revision, read, and approved the submitted version.
