# Supplementary material for: Comparison of plant diversity-carbon storage relationships along altitudinal gradients in temperate forests and shrublands
Source: Front Plant Sci. 2023 Aug 11;14:1120050. doi: 10.3389/fpls.2023.1120050 (PMC10453807; doi:10.3389/fpls.2023.1120050)
Supplement: Supplementary file 1 [file DataSheet_1.docx]

Supplementary Material

Comparison of plant diversity-carbon storage relationships along altitudinal gradients in temperate forests and shrublands

Shuaizhi Lu ^1,2^, Dou Zhang ^3^, Le Wang ^4^, Lei Dong ^5^, Changcheng Liu ^1,2^, Dongjie Hou ^6^, Guoping Chen ^1,2^, Xianguo Qiao ^1,2^, Yuyouting Wang ^7^, Ke Guo ^1,2*^

^1^State Key Laboratory of Vegetation and Environmental Change, Institute of Botany, Chinese Academy of Sciences, Beijing 100093, China

^2^University of Chinese Academy of Sciences, Beijing 100049, China

^3^Department of Environmental Science and Engineering, Fudan University, Shanghai 200438, China

^4^Institute of Ecological Protection and Restoration, Chinese Academy of Forestry, Beijing 100093, China.

^5^Institute of Water Resources for pastoral Area Ministry of Water Resources, Inner Mongolia 010000, China

^6^Inner Mongolia Agricultural University, Inner Mongolia 010019, China

^7^Yunnan Climate Center, Kunming 650034, China

*** Correspondence:**Ke Guo
guoke@ibcas.ac.cn

# Supplementary Tables

Supplementary Table 1. The allometric growth equations for tree and shrub species. Abbreviation: W_1_, stem biomass; W_2_, branch biomass; W_3,_ leaf biomass; W_4,_ root biomass; W, individual stand biomass; D, diameter at breast height (tree) and basal diameter (shrub); H, tree height.

| **Plant species** | **Regression models** | **Carbon content** |
| --- | --- | --- |
| ***tree*** |  |  |
| *Betula platyphylla* | W_1_=0.0642(D^2^H)^0.8734^ | 0.4890 |
|  | W_2_=0.0165(D^2^H)^0.7471^ | 0.5689 |
|  | W_3_=0.0054(D^2^H)^0.7486^ | 0.5068 |
|  | W_4_=0.0042(D^2^H)^1.0629^ | 0.4934 |
| *Populus davidiana* | W_1_=0.0859(D^2^H)^0.8001^ | 0.4844 |
|  | W_2_=0.0036(D^2^H)^0.8437^ | 0.4866 |
|  | W_3_=0.0291(D^2^H)^0.6136^ | 0.4960 |
|  | W_4_=0.0143(D^2^H)^0.8772^ | 0.4861 |
| *Quercus variabilis* | W_1_= 0.0440(D^2^H)^0.9418^ | 0.4792 |
|  | W_2_=0.0146(D^2^H)^0.9607^ | 0.4826 |
|  | W_3_=0.0058(D^2^H)^0.9015^ | 0.4767 |
|  | W_4_=0.0468(D^2^H)^0.7500^ | 0.4725 |
| *Ulmus pumila* | W_1_=0.0773(D^2^H)^0.9947^ | 0.4857 |
|  | W_2_=0.0814(D^2^H)^0.7510^ | 0.4848 |
|  | W_3_=0.0178(D^2^H)^0.7584^ | 0.4905 |
|  | W_4_=0.0772(D^2^H)^0.8176^ | 0.4853 |
| *Robinia pseudoacacia* | W_1_=0.0302(D^2^H)^0.9474^ | 0.4857 |
|  | W_2_=0.0040(D^2^H)^0.4848^ | 0.4848 |
|  | W_3_=0.0060(D^2^H)^0.4905^ | 0.4905 |
|  | W_4_=0.0119(D^2^H)^0.4853^ | 0.4853 |
| *Acer truncatum* | W_1_=0.0314(D^2^H)^0.9775^ | 0.4857 |
|  | W_2_=0.0069(D^2^H)^1.1030^ | 0.4848 |
|  | W_3_=0.0060(D^2^H)^0.8803^ | 0.4905 |
|  | W_4_=0.0468(D^2^H)^0.8939^ | 0.4853 |
| *Betula dahurica* | W_1_= 0.0842(D^2^H)^0.7965^ | 0.4890 |
|  | W_2_= 0.0033(D^2^H)^1.0630^ | 0.5046 |
|  | W_3_= 0.0035(D^2^H)^0.8603^ | 0.5068 |
|  | W_4_= 0.0269(D^2^H)^0.8222^ | 0.4934 |
| *Quercus mongolica* | W_1_=0.1131(D^2^H)^0.8631^ | 0.4792 |
|  | W_2_=0.0049(D^2^H)^1.1016^ | 0.4826 |
|  | W_3_=0.0156(D^2^H)^0.7295^ | 0.4767 |
|  | W_4_=0.0428(D^2^H)^0.8652^ | 0.4725 |
| *Populus × Canadensis,* *Populus cathayana,* *Populus simonii,* *Populus cathayana* | W_1_=0.0204(D^2^H)^0.9842^ | 0.4844 |
|  | W_2_=0.0019(D^2^H)^1.1198^ | 0.4866 |
|  | W_3_=0.0122(D^2^H)^0.7474^ | 0.4960 |
|  | W_4_=0.0079(D^2^H)^0.9596^ | 0.4861 |
| *Quercus dentate,* *Quercus aliena* | W_1_=0.0560(D^2^H)^0.9140^ | 0.4792 |
|  | W_2_=0.0080(D^2^H)^1.0370^ | 0.4826 |
|  | W_3_=0.0060(D^2^H)^0.8830^ | 0.4767 |
|  | W_4_=0.0720(D^2^H)^0.8760^ | 0.4725 |
| *Betula costata* | W_1_=0.1040(D^2^H)^0.7926^ | 0.4857 |
|  | W_2_=0.0087(D^2^H)^0.8855^ | 0.4848 |
|  | W_3_=0.0064(D^2^H)^0.7453^ | 0.4905 |
|  | W_4_=0.0155(D^2^H)^0.8805^ | 0.4853 |
| *Celtis bungeana,* *Pyrus ussuriensis,* *Juglans mandshurica,* *Morus mongolica,* *Broussonetia papyrifera,* *Pteroceltis tatarinowii,* *Diospyros lotus,* *Koelreuteria paniculata,* *Ulmus laciniata* | W_1_=0.0545(D^2^H)^0.8630^ | 0.4857 |
|  | W_2_=0.0155(D^2^H)^0.8737^ | 0.4848 |
|  | W_3_=0.0145(D^2^H)^0.7444^ | 0.4905 |
|  | W_4_=0.0307(D^2^H)^0.8270^ | 0.4853 |
| *Pterocarya stenoptera,* *Salix matsudana* | W_1_=0.0699(D_2_H)^0.8254^ | 0.4857 |
|  | W_2_=0.0267(D_2_H)^0.7207^ | 0.4848 |
|  | W_3_=0.0125(D_2_H)^0.6181^ | 0.4905 |
|  | W_4_=0.0363(D_2_H)^0.7529^ | 0.4853 |
| *Pinus tabuliformis* | W_1_=0.0634(D^2^H)^0.8189^ | 0.4958 |
|  | W_2_=0.0111(D^2^H)^0.9805^ | 0.5019 |
|  | W_3_=0.0185(D^2^H)^0.8195^ | 0.5158 |
|  | W_4_=0.0085(D^2^H)^0.9324^ | 0.5058 |
| *Larix principis-rupprechtii* | W_1_= 0.0428(D^2^H)^0.9432^ | 0.4971 |
|  | W_2_=0.0088(D^2^H)^0.09027^ | 0.5100 |
|  | W_3_= 0.0490(D^2^H)^0.6459^ | 0.5107 |
|  | W_4_= 0.0114(D^2^H)^0.9512^ | 0.5337 |
| *Picea wilsonii, Picea meyeri* | W_1_= 0.0408(D^2^H)^0.9020^ | 0.5009 |
|  | W_2_= 0.0953(D^2^H)^0.6714^ | 0.4963 |
|  | W_3_= 0.1049(D^2^H)^0.6249^ | 0.5205 |
|  | W_4_= 0.0221(D^2^H)^0.8509^ | 0.5257 |
| ***Shrub*** |  |  |
| *Lespedeza bicolor* | W=0.6700(D^2^H)^0.7280^ | 0.4897 |
| *Ostryopsis davidiana* | W=0.1280(D^2^H)^0.6930^ | 0.4897 |
| *Armeniaca sibirica* | W=0.2080(D^2^H)^0.7590^ | 0.4897 |
| *Cotinus coggygria* | W=0.1030(D^2^H)^0.8260^ | 0.4897 |
| *Spiraea trilobata* | W_1_=0.0243(D^2^H)^1.0987^ | 0.4897 |
|  | W_2_=0.3943(D^2^H)^0.9533^ |  |
|  | W_3_= 1.1857(D^2^H)^0.8847^ |  |
| *Spiraea pubescens* | W_1_=0.02433(D^2^H)^1.0957^ | 0.4897 |
|  | W_2_=0.3942(D^2^H)^0.9584^ |  |
|  | W_3_=1.1857(D^2^H)^0.0548^ |  |
| *Vitex negundo var.heterophylla* | W=0.0620+0.0410(D^2^H) | 0.4897 |
| *Ziziphus jujuba var. spinosa* | W=0.1210(D^2^H)^0.6070^ | 0.4897 |
| *Corylus heterophylla* | W=0.0486(D^2^H)^0.7953^ | 0.4897 |
| *Carpinus turczaninowii,* *Salix character,* *Artemisia sacrorum,* *Wikstroemia chamaedaphne,* *Leptopus chinensis,* *Amygdalus davidiana,* *Indigofera bungeana* | W_1_=0.0487+0.0028(D^2^H) | 0.4897 |
|  | W_2_=0.0669+0.0226(D^2^H) |  |
|  | W_3_=0.0119(0.0669+0.0226(D^2^H)+ 0.0487+0.0028(D^2^H))^0.6172^ |  |

Supplementary Table 2. The composite pSEM accounting for the direct and indirect standardized effects of mean annual temperature (MAT), soil moisture (SM), structural diversity (STRUC) and species diversity (SPED) on carbon storage in temperate forests. Significant levels are indicated *** (P<0.001), ** (P<0.01) and * (P<0.05).

| Response | Mediator | Predictor | Effect | Path label | Std.Est | Std.Error | Z-value | P-value |
| --- | --- | --- | --- | --- | --- | --- | --- | --- |
| Carbon Storage | ---- | STRUC | Direct | a | 0.369 | 0.164 | 4.509 | 0.000 *** |
| Carbon Storage | ---- | SPED | Direct | b | 0.251 | 0.243 | 3.256 | 0.002 ** |
| Carbon Storage | ---- | MAT | Direct | c | -0.185 | 0.087 | -2.124 | 0.036 * |
| Carbon Storage | ---- | SM | Direct | d | 0.131 | 0.084 | 1.546 | 0.125 |
| STRUC | ---- | SM | Direct | e | 0.124 | 0.048 | 1.286 | 0.201 |
| STRUC | ---- | MAT | Direct | f | -0.305 | 0.048 | -3.173 | 0.002 ** |
| SPED | ---- | SM | Direct | g | 0.198 | 0.032 | 1.938 | 0.055 |
| SPED | ---- | MAT | Direct | h | 0.091 | 0.032 | 0.889 | 0.376 |
| SM | ---- | MAT | Direct | i | -0.412 | 0.086 | -4.806 | 0.000 *** |
| Carbon Storage | STRUC | MAT | Indirect | a*f | -0.113 |  |  |  |
| Carbon Storage | SPED | MAT | Indirect | b*h |  |  |  |  |
| Carbon Storage | SM | MAT | Indirect | d*i |  |  |  |  |
| Carbon Storage | STRUC | SM | Indirect | a*e |  |  |  |  |
| Carbon Storage | SPED | SM | Indirect | b*g |  |  |  |  |
| Carbon Storage | STRUC+SPED+SM | MAT | Total | c+(a*f)+(b*h)+(d*i) | -0.298 |  |  |  |
| Carbon Storage | STRUC | SM | Total | d+(a*e)+(b*g) |  |  |  |  |

Supplementary Table 3. The composite pSEM accounting for the direct and indirect standardized effects of mean annual temperature (MAT), soil moisture (SM), structural diversity (STRUC) and species diversity (SPED) on carbon storage in temperate forests at high altitude. Significant levels are indicated *** (P<0.001), ** (P<0.01) and * (P<0.05).

| Response | Mediator | Predictor | Effect | Path label | Std.Est | Std.Error | Z-value | P-value |
| --- | --- | --- | --- | --- | --- | --- | --- | --- |
| Carbon Storage | ---- | STRUC | Direct | a | 0.616 | 0.228 | 4.739 | 0.000 *** |
| Carbon Storage | ---- | SPED | Direct | b | 0.323 | 0.633 | 2.271 | 0.030 * |
| Carbon Storage | ---- | MAT | Direct | c | 0.184 | 0.092 | 1.421 | 0.165 |
| Carbon Storage | ---- | SM | Direct | d | -0.208 | 0.126 | -1.454 | 0.155 |
| STRUC | ---- | SM | Direct | e | 0.084 | 0.083 | 0.510 | 0.613 |
| STRUC | ---- | MAT | Direct | f | -0.144 | 0.067 | -0.877 | 0.386 |
| SPED | ---- | SM | Direct | g | 0.433 | 0.030 | 2.874 | 0.007 ** |
| SPED | ---- | MAT | Direct | h | -0.026 | 0.024 | -0.175 | 0.862 |
| SM | ---- | MAT | Direct | i | -0.080 | 0.131 | -0.489 | 0.628 |
| Carbon Storage | STRUC | MAT | Indirect | a*f |  |  |  |  |
| Carbon Storage | SPED | MAT | Indirect | b*h |  |  |  |  |
| Carbon Storage | SM | MAT | Indirect | d*i |  |  |  |  |
| Carbon Storage | STRUC | SM | Indirect | a*e |  |  |  |  |
| Carbon Storage | SPED | SM | Indirect | b*g | 0.140 |  |  |  |
| Carbon Storage | STRUC+SPED+SM | MAT | Total | c+(a*f)+(b*h)+(d*i) |  |  |  |  |
| Carbon Storage | STRUC | SM | Total | d+(a*e)+(b*g) | 0.140 |  |  |  |

Supplementary Table 4. The composite pSEM accounting for the direct and indirect standardized effects of mean annual temperature (MAT), soil moisture (SM), structural diversity (STRUC) and species diversity (SPED) on carbon storage in temperate forests at middle altitude. Significant levels are indicated *** (P<0.001), ** (P<0.01) and * (P<0.05).

| Response | Mediator | Predictor | Effect | Path label | Std.Est | Std.Error | Z-value | P-value |
| --- | --- | --- | --- | --- | --- | --- | --- | --- |
| Carbon Storage | ---- | STRUC | Direct | a | 0.489 | 0.254 | 3.164 | 0.004 ** |
| Carbon Storage | ---- | SPED | Direct | b | 0.060 | 0.428 | 0.419 | 0.678 |
| Carbon Storage | ---- | MAT | Direct | c | -0.159 | 0.329 | -1.097 | 0.282 |
| Carbon Storage | ---- | SM | Direct | d | 0.333 | 0.171 | 2.495 | 0.019 * |
| STRUC | ---- | SPED | Direct | e | 0.329 | 0.274 | 2.187 | 0.037 * |
| STRUC | ---- | MAT | Direct | f | -0.414 | 0.207 | -2.754 | 0.010 * |
| SPED | ---- | SM | Direct | g | 0.236 | 0.075 | 1.348 | 0.188 |
| SPED | ---- | MAT | Direct | h | -0.059 | 0.133 | -0.338 | 0.738 |
| SM | ---- | MAT | Direct | i | -0.127 | 0.310 | -0.722 | 0.476 |
| Carbon Storage | STRUC | MAT | Indirect | a*f | -0.202 |  |  |  |
| Carbon Storage | SPED | MAT | Indirect | b*h |  |  |  |  |
| Carbon Storage | SM | MAT | Indirect | d*i |  |  |  |  |
| Carbon Storage | STRUC | SPED | Indirect | a*e | 0.161 |  |  |  |
| Carbon Storage | SPED | SM | Indirect | b*g |  |  |  |  |
| Carbon Storage | STRUC+SPED+SM | MAT | Total | c+(a*f)+(b*h)+(d*i) | -0.202 |  |  |  |
| Carbon Storage | STRUC | SM | Total | d+(a*e)+(b*g) | 0.333 |  |  |  |

Supplementary Table 5. The composite pSEM accounting for the direct and indirect standardized effects of mean annual temperature (MAT), soil moisture (SM), structural diversity (STRUC) and species diversity (SPED) on carbon storage in temperate forests at low altitude. Significant levels are indicated *** (P<0.001), ** (P<0.01) and * (P<0.05).

| Response | Mediator | Predictor | Effect | Path label | Std.Est | Std.Error | Z-value | P-value |
| --- | --- | --- | --- | --- | --- | --- | --- | --- |
| Carbon Storage | ---- | STRUC | Direct | a | 0.338 | 0.292 | 2.604 | 0.013 * |
| Carbon Storage | ---- | SPED | Direct | b | 0.338 | 0.284 | 2.732 | 0.010 * |
| Carbon Storage | ---- | MAT | Direct | c | -0.411 | 0.621 | -2.715 | 0.010 * |
| Carbon Storage | ---- | SM | Direct | d | -0.135 | 0.163 | -0.871 | 0.389 |
| STRUC | ---- | SM | Direct | e | 0.282 | 0.087 | 1.508 | 0.140 |
| STRUC | ---- | MAT | Direct | f | -0.076 | 0.341 | -0.404 | 0.688 |
| SPED | ---- | SM | Direct | g | 0.033 | 0.090 | 0.170 | 0.866 |
| SPED | ---- | MAT | Direct | h | -0.128 | 0.350 | -0.655 | 0.516 |
| SM | ---- | MAT | Direct | i | -0.589 | 0.498 | -4.613 | 0.000 *** |
| Carbon Storage | STRUC | MAT | Indirect | a*f |  |  |  |  |
| Carbon Storage | SPED | MAT | Indirect | b*h |  |  |  |  |
| Carbon Storage | SM | MAT | Indirect | d*i |  |  |  |  |
| Carbon Storage | STRUC | SM | Indirect | a*e |  |  |  |  |
| Carbon Storage | SPED | SM | Indirect | b*g |  |  |  |  |
| Carbon Storage | STRUC+SPED+SM | MAT | Total | c+(a*f)+(b*h)+(d*i) | -0.411 |  |  |  |
| Carbon Storage | STRUC | SM | Total | d+(a*e)+(b*g) |  |  |  |  |

Supplementary Table 6. The composite pSEM accounting for the direct and indirect standardized effects of mean annual temperature (MAT), soil moisture (SM), structural diversity (STRUC) and species diversity (SPED) on carbon storage in temperate shrublands. Significant levels are indicated *** (P<0.001), ** (P<0.01) and * (P<0.05).

| Response | Mediator | Predictor | Effect | Path label | Std.Est | Std.Error | Z-value | P-value |
| --- | --- | --- | --- | --- | --- | --- | --- | --- |
| Carbon Storage | ---- | STRUC | Direct | a | 0.216 | 0.181 | 3.022 | 0.003 ** |
| Carbon Storage | ---- | SPED | Direct | b | 0.514 | 0.109 | 7.810 | 0.000 *** |
| Carbon Storage | ---- | MAT | Direct | c | 0.233 | 0.079 | 2.947 | 0.004 ** |
| Carbon Storage | ---- | SM | Direct | d | 0.038 | 0.065 | 0.587 | 0.558 |
| STRUC | ---- | SM | Direct | e | 0.128 | 0.034 | 1.484 | 0.140 |
| STRUC | ---- | MAT | Direct | f | 0.324 | 0.034 | 3.764 | 0.000 *** |
| SPED | ---- | SM | Direct | g | 0.138 | 0.052 | 1.606 | 0.111 |
| SPED | ---- | MAT | Direct | h | 0.379 | 0.061 | 3.731 | 0.000 *** |
| SM | ---- | MAT | Direct | i | -0.265 | 0.084 | -3.156 | 0.002 |
| Carbon Storage | STRUC | MAT | Indirect | a*f | 0.070 |  |  |  |
| Carbon Storage | SPED | MAT | Indirect | b*h | 0.195 |  |  |  |
| Carbon Storage | SM | MAT | Indirect | d*i |  |  |  |  |
| Carbon Storage | STRUC | SM | Indirect | a*e |  |  |  |  |
| Carbon Storage | SPED | SM | Indirect | b*g |  |  |  |  |
| Carbon Storage | STRUC+SPED+SM | MAT | Total | c+(a*f)+(b*h)+(d*i) | 0.497 |  |  |  |
| Carbon Storage | STRUC | SM | Total | d+(a*e)+(b*g) |  |  |  |  |

Supplementary Table 7. The composite pSEM accounting for the direct and indirect standardized effects of mean annual temperature (MAT), soil moisture (SM), structural diversity (STRUC) and species diversity (SPED) on carbon storage in temperate shrublands at high altitude. Significant levels are indicated *** (P<0.001), ** (P<0.01) and * (P<0.05).

| Response | Mediator | Predictor | Effect | Path label | Std.Est | Std.Error | Z-value | P-value |
| --- | --- | --- | --- | --- | --- | --- | --- | --- |
| Carbon Storage | ---- | STRUC | Direct | a | 0.443 | 0.258 | 3.206 | 0.003 ** |
| Carbon Storage | ---- | SPED | Direct | b | 0.510 | 0.216 | 4.383 | 0.000 *** |
| Carbon Storage | ---- | MAT | Direct | c | 0.097 | 0.112 | 0.687 | 0.496 |
| Carbon Storage | ---- | SM | Direct | d | 0.028 | 0.130 | 0.243 | 0.809 |
| STRUC | ---- | SM | Direct | e | 0.169 | 0.090 | 1.143 | 0.260 |
| STRUC | ---- | MAT | Direct | f | 0.405 | 0.063 | 2.745 | 0.009 ** |
| SPED | ---- | SM | Direct | g | -0.115 | 0.095 | -0.747 | 0.460 |
| SPED | ---- | MAT | Direct | h | 0.306 | 0.081 | 1.632 | 0.111 |
| SM | ---- | MAT | Direct | i | -0.201 | 0.107 | -1.312 | 0.197 |
| Carbon Storage | STRUC | MAT | Indirect | a*f | 0.179 |  |  |  |
| Carbon Storage | SPED | MAT | Indirect | b*h |  |  |  |  |
| Carbon Storage | SM | MAT | Indirect | d*i |  |  |  |  |
| Carbon Storage | STRUC | SM | Indirect | a*e |  |  |  |  |
| Carbon Storage | SPED | SM | Indirect | b*g |  |  |  |  |
| Carbon Storage | STRUC+SPED+SM | MAT | Total | c+(a*f)+(b*h)+(d*i) | 0.179 |  |  |  |
| Carbon Storage | STRUC | SM | Total | d+(a*e)+(b*g) |  |  |  |  |

Supplementary Table 8. TThe composite pSEM accounting for the direct and indirect standardized effects of mean annual temperature (MAT), soil moisture (SM), structural diversity (STRUC) and species diversity (SPED) on carbon storage in temperate shrublands at middle altitude. Significant levels are indicated *** (P<0.001), ** (P<0.01) and * (P<0.05).

| Response | Mediator | Predictor | Effect | Path label | Std.Est | Std.Error | Z-value | P-value |
| --- | --- | --- | --- | --- | --- | --- | --- | --- |
| Carbon Storage | ---- | STRUC | Direct | a | 0.063 | 0.372 | 0.493 | 0.625 |
| Carbon Storage | ---- | SPED | Direct | b | 0.563 | 0.220 | 4.335 | 0.000 *** |
| Carbon Storage | ---- | MAT | Direct | c | 0.220 | 0.412 | 1.864 | 0.070 |
| Carbon Storage | ---- | SM | Direct | d | -0.041 | 0.105 | -0.342 | 0.734 |
| STRUC | ---- | SPED | Direct | e | 0.416 | 0.080 | 3.016 | 0.004 ** |
| STRUC | ---- | MAT | Direct | f | 0.018 | 0.169 | 0.130 | 0.897 |
| SPED | ---- | SM | Direct | g | 0.302 | 0.074 | 2.102 | 0.042 * |
| SPED | ---- | MAT | Direct | h | 0.084 | 0.307 | 0.566 | 0.574 |
| SM | ---- | MAT | Direct | i | -0.076 | 0.593 | -0.513 | 0.611 |
| Carbon Storage | STRUC | MAT | Indirect | a*f |  |  |  |  |
| Carbon Storage | SPED | MAT | Indirect | b*h |  |  |  |  |
| Carbon Storage | SM | MAT | Indirect | d*i |  |  |  |  |
| Carbon Storage | STRUC | SPED | Indirect | a*e |  |  |  |  |
| Carbon Storage | SPED | SM | Indirect | b*g | 0.170 |  |  |  |
| Carbon Storage | STRUC+SPED+SM | MAT | Total | c+(a*f)+(b*h)+(d*i) |  |  |  |  |
| Carbon Storage | STRUC | SM | Total | d+(a*e)+(b*g) | 0.170 |  |  |  |

Supplementary Table 9. The composite pSEM accounting for the direct and indirect standardized effects of mean annual temperature (MAT), soil moisture (SM), structural diversity (STRUC) and species diversity (SPED) on carbon storage in temperate shrublands at low altitude. Significant levels are indicated *** (P<0.001), ** (P<0.01) and * (P<0.05).

| Response | Mediator | Predictor | Effect | Path label | Std.Est | Std.Error | Z-value | P-value |
| --- | --- | --- | --- | --- | --- | --- | --- | --- |
| Carbon Storage | ---- | STRUC | Direct | a | 0.126 | 0.713 | 1.432 | 0.160 |
| Carbon Storage | ---- | SPED | Direct | b | 0.683 | 0.123 | 6.991 | 0.000 *** |
| Carbon Storage | ---- | MAT | Direct | c | 0.218 | 0.468 | 1.958 | 0.057 |
| Carbon Storage | ---- | SM | Direct | d | 0.294 | 0.093 | 2.616 | 0.013 * |
| STRUC | ---- | SM | Direct | e | -0.066 | 0.019 | -0.361 | 0.720 |
| STRUC | ---- | MAT | Direct | f | -0.009 | 0.095 | -0.051 | 0.960 |
| SPED | ---- | SM | Direct | g | 0.461 | 0.108 | 2.801 | 0.008 ** |
| SPED | ---- | MAT | Direct | h | 0.436 | 0.551 | 2.652 | 0.011 * |
| SM | ---- | MAT | Direct | i | -0.522 | 0.673 | -3.967 | 0.000 *** |
| Carbon Storage | STRUC | MAT | Indirect | a*f |  |  |  |  |
| Carbon Storage | SPED | MAT | Indirect | b*h | 0.298 |  |  |  |
| Carbon Storage | SM | MAT | Indirect | d*i | -0.154 |  |  |  |
| Carbon Storage | STRUC | SM | Indirect | a*e |  |  |  |  |
| Carbon Storage | SPED | SM | Indirect | b*g | 0.315 |  |  |  |
| Carbon Storage | STRUC+SPED+SM | MAT | Total | c+(a*f)+(b*h)+(d*i) | 0.144 |  |  |  |
| Carbon Storage | STRUC | SM | Total | d+(a*e)+(b*g) | 0.609 |  |  |  |

Supplementary Table 10. The direct effects of species diversity, structural diversity, and altitude on forest function (biomass, carbon storage or productivity) in related studies. “+”, “−”, “-” indicate positive, negative and non-significant effect. The numbers are standard correlation coefficients. Abbreviations: AGC, aboveground carbon storage; AGB, aboveground biomass; TC, total carbon storage including plant and soil; TB, above and belowground biomass; ANPP, aboveground net primary productivity.

| Location (biome) | Forest function | Species  diversity | Structural  diversity | Altitude | Ref. |
| --- | --- | --- | --- | --- | --- |
| Neotropics (Tropical forests) | AGB | +(0.20) | +(0.26) |  | (Poorter et al., 2015) |
| Hainan Island (Tropical forests) | AGB | - | +(0.86) |  | (Ali et al., 2019) |
| Hainan Island (Tropical forests) | AGC | - | +(0.44) | +(0.58) | (Wen et al., 2022) |
| Eastern China (Subtropical forests) | AGC | −(0.23) | +(0.56) |  | (Ali et al., 2016) |
| China (Subtropical forests) | TC | +(0.75) |  |  | (Yan et al., 2022) |
| Tiantong National Forest Park (Subtropical forests) | AGB | - | +(0.51) |  | (Ren et al., 2021) |
| Northern Iran (Temperate forests) | AGC | - | +(0.26) | +(0.25) | (Kazempour Larsary et al., 2021) |
| Northeast China (Temperate forests) | AGC |  | +(0.36) | +(0.18) | (Yuan et al., 2021) |
| Southeast Australia (Temperate forests) | AGC | +(0.18) | +(0.43) |  | (Aponte et al., 2020) |
| Northern Pakistan (Temperate forests) | AGB |  | +(0.10) | −(0.33) | (Ullah et al., 2021) |
| Northern Ethiopia (Dry Afromontane forests) | AGC | −(0.13) | +(0.88) | −(0.10) | (Tetemke et al., 2021) |
| China (All forests) | ANPP | +(0.31) |  |  | (Chen et al., 2018) |
| Yellow River basin (Subtropical shrublands) | AGB | +(0.27) |  |  | (Yi et al., 2021) |
| Yellow River basin (Temperate shrublands) | AGB | +(0.38) | +(0.26) |  | (Yi et al., 2021) |
| Northern China (Shrublands with low water availability) | TB | +(0.26) |  |  | (Guo et al., 2019) |
| Northern China (Shrublands with high water availability) | TB | −(0.24) |  |  | (Guo et al., 2019) |

# Supplementary Figures


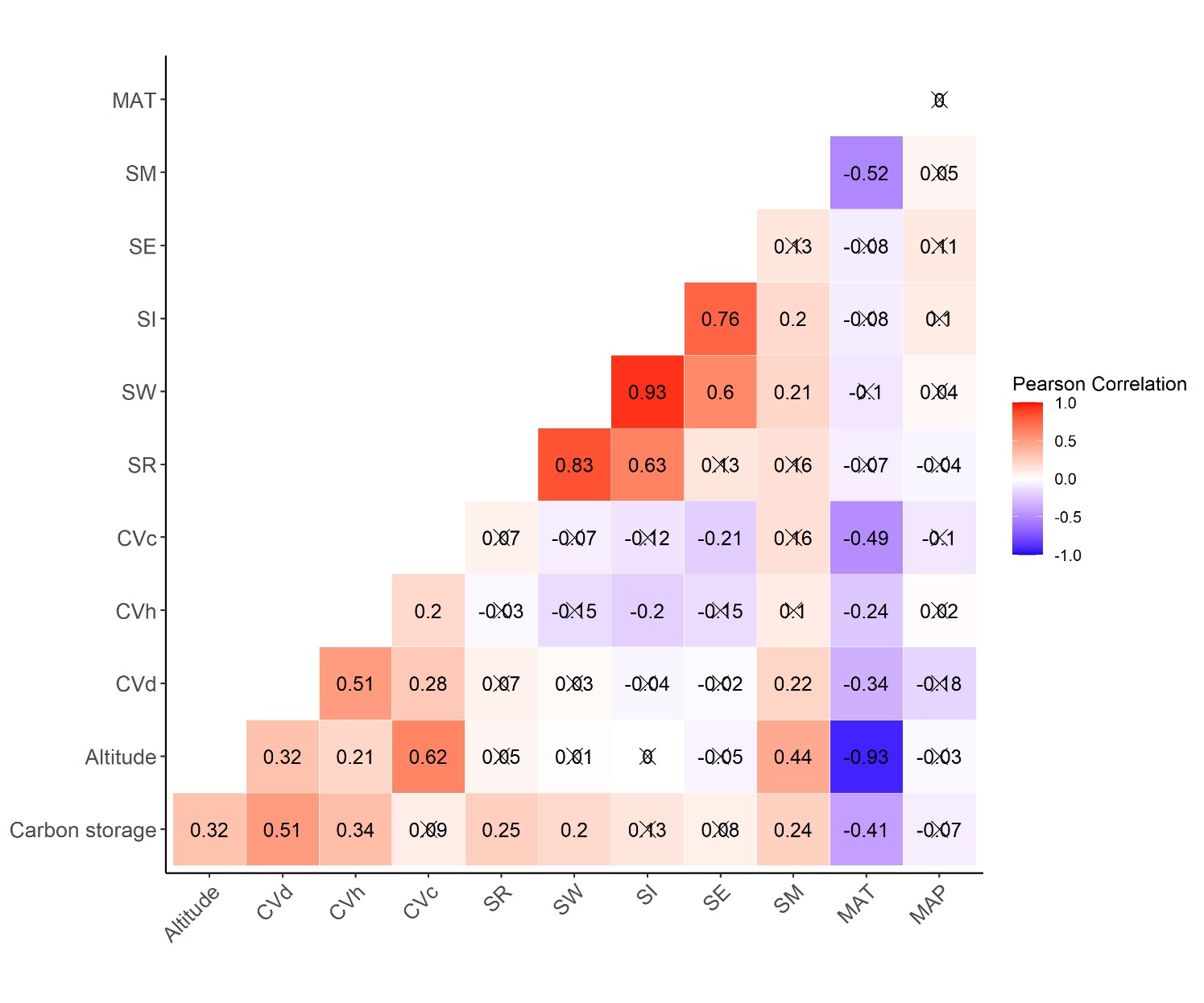


**Supplementary Figure 1.** Pearson correlation of selecting optimal variables for pSEM **in forests**. Values in the matrix represent Pearson correlation coefficients. The cross symbol represents non-significant (p >0.05). Abbreviations: CV*_d_*, coefficient of variation in diameter at breast height (for trees) or basal diameter (for shrubs) ; CV*_h_*, coefficient of variation in tree height; CV*_c_*, coefficient of variation in crown width; SR, species richness index; SW, Shannon’s Wiener index; SI, Simpson index; SE, Shannon evenness index; SM, soil moisture; MAT, mean annual temperature; MAP, mean annual precipitation.


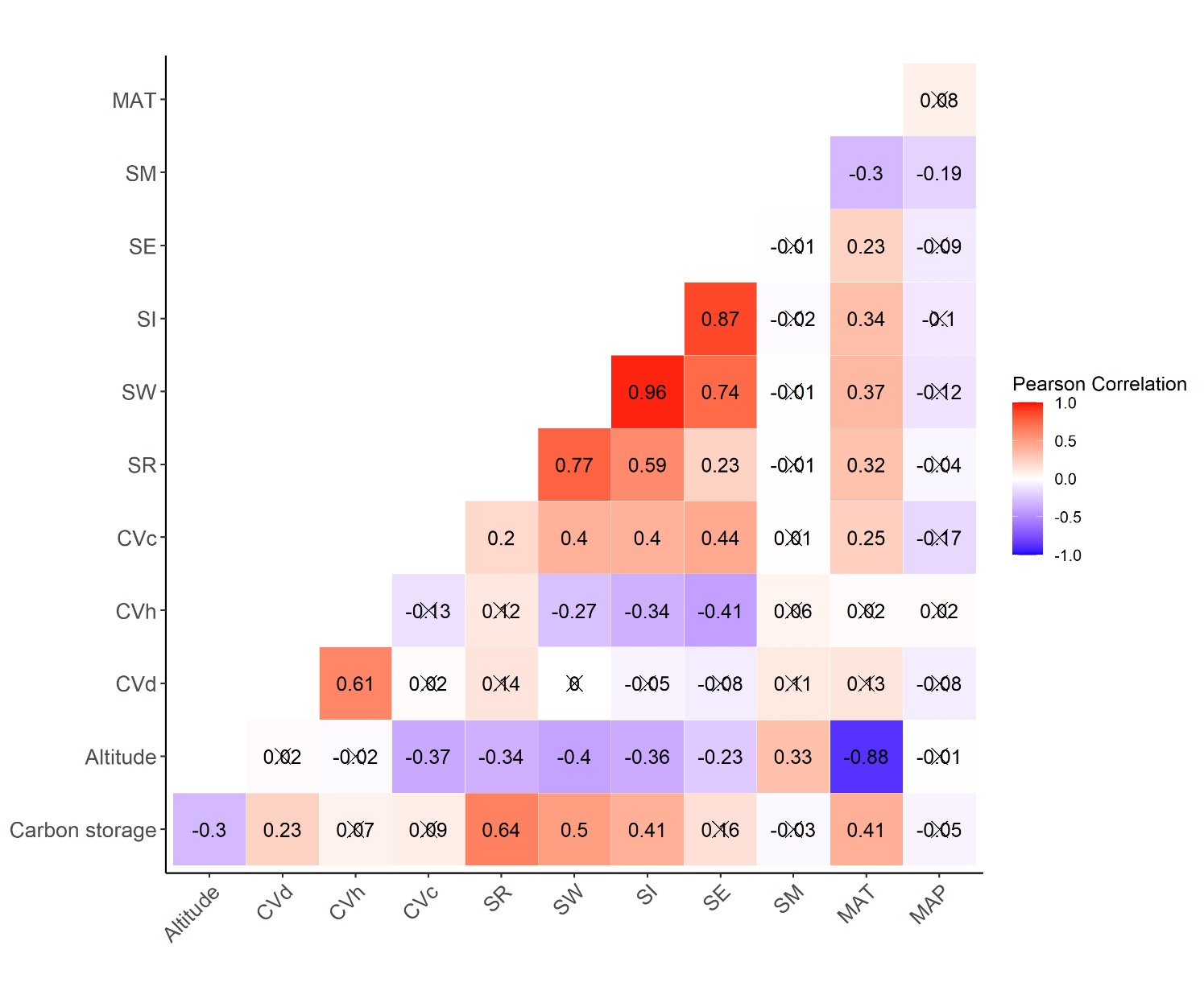


**Supplementary Figure 2.** Pearson correlation of selecting optimal variables for pSEM **in shrublands**. Values in the matrix represent Pearson correlation coefficients. The cross symbol represents non-significant (p>0.05). Abbreviations: CV*_d_*, coefficient of variation in diameter at breast height (for trees) or basal diameter (for shrubs) ; CV*_h_*, coefficient of variation in tree height; CV*_c_*, coefficient of variation in crown width; SR, species richness index; SW, Shannon’s Wiener index; SI, Simpson index; SE, Shannon evenness index; SM, soil moisture; MAT, mean annual temperature; MAP, mean annual precipitation.

# Codes

getwd()

setwd("E:/data process/Finaldata/plot3")

install.packages("piecewiseSEM")

install.packages("readxl")

install.packages("TH.data")

install.packages("ggplot2")

install.packages("readr")

install.packages("lavaan")

install.packages("semPlot")

install.packages("lme4")

install.packages("nlme")

install.packages("QuantPsyc")

install.packages("boot")

install.packages("dplyr")

install.packages("purrr")

install.packages("MASS")

library(lme4)

library(nlme)

library(piecewiseSEM)

library(QuantPsyc)

library(readxl)

library(readr)

library(lavaan)

library(semPlot)

library(Matrix)

library(boot)

library(dplyr)

library(MASS)

library(purrr)

########################### Composite PSEM ############################

############## Forest ###############

d1 <- read_excel("plot0410.xls", sheet = "forest_st")

#结构多样性变量

model1 <- lm(C ~ CVd + CVh, d1)

coefs(model1, standardize = 'scale')

beta_CVd <- summary(model1)$coefficients[2, 1]

beta_CVh <- summary(model1)$coefficients[3, 1]

STRUC <- beta_CVd * d1$CVd + beta_CVh * d1$CVh

d1$STRUC <- STRUC

summary(lm(C ~ STRUC, d1))

coefs(lm(C ~ STRUC, d1))

#物种多样性变量

model2 <- lm(C ~ SR + SW +SI, d1)

coefs(model2, standardize = 'scale')

beta_SR <- summary(model2)$coefficients[2, 1]

beta_SW <- summary(model2)$coefficients[3, 1]

beta_SI <- summary(model2)$coefficients[4, 1]

SP <- beta_SR * d1$SR + beta_SW * d1$SW + beta_SI * d1$SI

d1$SP <- SP

summary(lm(C ~ SP, d1))

coefs(lm(C ~ SP, d1))

microbe.list <- list(

lm(C ~ STRUC + SP + MAT + SM, d1),

lm(STRUC ~ SM + MAT, d1),

lm(SP ~ SM + MAT, d1),

lm(SM ~ MAT, d1)

)

microbe.psem <- as.psem(microbe.list)

(nem.summary <-summary(microbe.psem, .progressBar = F))

plot(microbe.psem)

############## high Forest ###############

d <- read_excel("plot0410.xls", sheet = "forest_st")

d1 <- d[ which(d$ALG =="3"), ]

#结构多样性变量

model1 <- lm(C ~ CVd + CVh, d1)

coefs(model1, standardize = 'scale')

beta_CVd <- summary(model1)$coefficients[2, 1]

beta_CVh <- summary(model1)$coefficients[3, 1]

STRUC <- beta_CVd * d1$CVd + beta_CVh * d1$CVh

d1$STRUC <- STRUC

summary(lm(C ~ STRUC, d1))

coefs(lm(C ~ STRUC, d1))

#物种多样性变量

model2 <- lm(C ~ SR + SW +SI, d1)

coefs(model2, standardize = 'scale')

beta_SR <- summary(model2)$coefficients[2, 1]

beta_SW <- summary(model2)$coefficients[3, 1]

beta_SI <- summary(model2)$coefficients[4, 1]

SP <- beta_SR * d1$SR + beta_SW * d1$SW + beta_SI * d1$SI

d1$SP <- SP

summary(lm(C ~ SP, d1))

coefs(lm(C ~ SP, d1))

microbe.list <- list(

lm(C ~ STRUC + SP + MAT + SM, d1),

lm(STRUC ~ SM + MAT, d1),

lm(SP ~ SM + MAT, d1),

lm(SM ~ MAT, d1)

)

microbe.psem <- as.psem(microbe.list)

(nem.summary <-summary(microbe.psem, .progressBar = F))

plot(microbe.psem)

############## middle Forest ###############

d <- read_excel("plot0410.xls", sheet = "forest_st")

d1 <- d[ which(d$ALG =="2"), ]

#结构多样性变量

model1 <- lm(C ~ CVd + CVh, d1)

coefs(model1, standardize = 'scale')

beta_CVd <- summary(model1)$coefficients[2, 1]

beta_CVh <- summary(model1)$coefficients[3, 1]

STRUC <- beta_CVd * d1$CVd + beta_CVh * d1$CVh

d1$STRUC <- STRUC

summary(lm(C ~ STRUC, d1))

coefs(lm(C ~ STRUC, d1))

#物种多样性变量

model2 <- lm(C ~ SR + SW +SI, d1)

coefs(model2, standardize = 'scale')

beta_SR <- summary(model2)$coefficients[2, 1]

beta_SW <- summary(model2)$coefficients[3, 1]

beta_SI <- summary(model2)$coefficients[4, 1]

SP <- beta_SR * d1$SR + beta_SW * d1$SW + beta_SI * d1$SI

d1$SP <- SP

summary(lm(C ~ SP, d1))

coefs(lm(C ~ SP, d1))

microbe.list <- list(

lm(C ~ STRUC + SP + MAT + SM, d1),

lm(STRUC ~ SP + MAT, d1),

lm(SP ~ SM + MAT, d1),

lm(SM ~ MAT, d1)

)

microbe.psem <- as.psem(microbe.list)

(nem.summary <-summary(microbe.psem, .progressBar = F))

plot(microbe.psem)

############## low Forest ###############

d <- read_excel("plot0410.xls", sheet = "forest_st")

d1 <- d[ which(d$ALG =="1"), ]

#结构多样性变量

model1 <- lm(C ~ CVd + CVh, d1)

coefs(model1, standardize = 'scale')

beta_CVd <- summary(model1)$coefficients[2, 1]

beta_CVh <- summary(model1)$coefficients[3, 1]

STRUC <- beta_CVd * d1$CVd + beta_CVh * d1$CVh

d1$STRUC <- STRUC

summary(lm(C ~ STRUC, d1))

coefs(lm(C ~ STRUC, d1))

#物种多样性变量

model2 <- lm(C ~ SR + SW +SI, d1)

coefs(model2, standardize = 'scale')

beta_SR <- summary(model2)$coefficients[2, 1]

beta_SW <- summary(model2)$coefficients[3, 1]

beta_SI <- summary(model2)$coefficients[4, 1]

SP <- beta_SR * d1$SR + beta_SW * d1$SW + beta_SI * d1$SI

d1$SP <- SP

summary(lm(C ~ SP, d1))

coefs(lm(C ~ SP, d1))

microbe.list <- list(

lm(C ~ STRUC + SP + MAT + SM, d1),

lm(STRUC ~ SM + MAT, d1),

lm(SP ~ SM + MAT, d1),

lm(SM ~ MAT, d1)

)

microbe.psem <- as.psem(microbe.list)

(nem.summary <-summary(microbe.psem, .progressBar = F))

plot(microbe.psem)

############## Shrub ###############

d1 <- read_excel("plot0410.xls", sheet = "shrub_st")

#结构多样性变量

model1 <- lm(C ~ CVd + CVh, d1)

coefs(model1, standardize = 'scale')

beta_CVd <- summary(model1)$coefficients[2, 1]

beta_CVh <- summary(model1)$coefficients[3, 1]

STRUC <- beta_CVd * d1$CVd + beta_CVh * d1$CVh

d1$STRUC <- STRUC

summary(lm(C ~ STRUC, d1))

coefs(lm(C ~ STRUC, d1))

#物种多样性变量

model2 <- lm(C ~ SR + SW +SI, d1)

coefs(model2, standardize = 'scale')

beta_SR <- summary(model2)$coefficients[2, 1]

beta_SW <- summary(model2)$coefficients[3, 1]

beta_SI <- summary(model2)$coefficients[4, 1]

SP <- beta_SR * d1$SR + beta_SW * d1$SW + beta_SI * d1$SI

d1$SP <- SP

summary(lm(C ~ SP, d1))

coefs(lm(C ~ SP, d1))

microbe.list <- list(

lm(C ~ STRUC + SP + MAT + SM, d1),

lm(STRUC ~ SM + MAT, d1),

lm(SP ~ SM + MAT, d1),

lm(SM ~ MAT, d1)

)

microbe.psem <- as.psem(microbe.list)

(nem.summary <-summary(microbe.psem, .progressBar = F))

plot(microbe.psem)

############## high Shrub ###############

d <- read_excel("plot0410.xls", sheet = "shrub_st")

d1 <- d[ which(d$ALG =="3"), ]

#结构多样性变量

model1 <- lm(C ~ CVd + CVh, d1)

coefs(model1, standardize = 'scale')

beta_CVd <- summary(model1)$coefficients[2, 1]

beta_CVh <- summary(model1)$coefficients[3, 1]

STRUC <- beta_CVd * d1$CVd + beta_CVh * d1$CVh

d1$STRUC <- STRUC

summary(lm(C ~ STRUC, d1))

coefs(lm(C ~ STRUC, d1))

#物种多样性变量

model2 <- lm(C ~ SR + SW +SI, d1)

coefs(model2, standardize = 'scale')

beta_SR <- summary(model2)$coefficients[2, 1]

beta_SW <- summary(model2)$coefficients[3, 1]

beta_SI <- summary(model2)$coefficients[4, 1]

SP <- beta_SR * d1$SR + beta_SW * d1$SW + beta_SI * d1$SI

d1$SP <- SP

summary(lm(C ~ SP, d1))

coefs(lm(C ~ SP, d1))

microbe.list <- list(

lm(C ~ STRUC + SP + MAT + SM, d1),

lm(STRUC ~ SM + MAT, d1),

lm(SP ~ SM + MAT, d1),

lm(SM ~ MAT, d1)

)

microbe.psem <- as.psem(microbe.list)

(nem.summary <-summary(microbe.psem, .progressBar = F))

plot(microbe.psem)

############## middle_Shrub ###############

d <- read_excel("plot0410.xls", sheet = "shrub_st")

d1 <- d[ which(d$ALG =="2"), ]

#结构多样性变量

model1 <- lm(C ~ CVd + CVh, d1)

coefs(model1, standardize = 'scale')

beta_CVd <- summary(model1)$coefficients[2, 1]

beta_CVh <- summary(model1)$coefficients[3, 1]

STRUC <- beta_CVd * d1$CVd + beta_CVh * d1$CVh

d1$STRUC <- STRUC

summary(lm(C ~ STRUC, d1))

coefs(lm(C ~ STRUC, d1))

#物种多样性变量

model2 <- lm(C ~ SR + SW +SI, d1)

coefs(model2, standardize = 'scale')

beta_SR <- summary(model2)$coefficients[2, 1]

beta_SW <- summary(model2)$coefficients[3, 1]

beta_SI <- summary(model2)$coefficients[4, 1]

SP <- beta_SR * d1$SR + beta_SW * d1$SW + beta_SI * d1$SI

d1$SP <- SP

summary(lm(C ~ SP, d1))

coefs(lm(C ~ SP, d1))

microbe.list <- list(

lm(C ~ STRUC + SP + MAT + SM, d1),

lm(STRUC ~ SP + MAT, d1),

lm(SP ~ SM + MAT, d1),

lm(SM ~ MAT, d1)

)

microbe.psem <- as.psem(microbe.list)

(nem.summary <-summary(microbe.psem, .progressBar = F))

plot(microbe.psem)

############## low_Shrub ###############

d <- read_excel("plot0410.xls", sheet = "shrub_st")

d1 <- d[ which(d$ALG =="1"), ]

#结构多样性变量

model1 <- lm(C ~ CVd + CVh, d1)

coefs(model1, standardize = 'scale')

beta_CVd <- summary(model1)$coefficients[2, 1]

beta_CVh <- summary(model1)$coefficients[3, 1]

STRUC <- beta_CVd * d1$CVd + beta_CVh * d1$CVh

d1$STRUC <- STRUC

summary(lm(C ~ STRUC, d1))

coefs(lm(C ~ STRUC, d1))

#物种多样性变量

model2 <- lm(C ~ SR + SW +SI, d1)

coefs(model2, standardize = 'scale')

beta_SR <- summary(model2)$coefficients[2, 1]

beta_SW <- summary(model2)$coefficients[3, 1]

beta_SI <- summary(model2)$coefficients[4, 1]

SP <- beta_SR * d1$SR + beta_SW * d1$SW + beta_SI * d1$SI

d1$SP <- SP

summary(lm(C ~ SP, d1))

coefs(lm(C ~ SP, d1))

microbe.list <- list(

lm(C ~ STRUC + SP + MAT + SM, d1),

lm(STRUC ~ SM + MAT, d1),

lm(SP ~ SM + MAT, d1),

lm(SM ~ MAT, d1)

)

microbe.psem <- as.psem(microbe.list)

(nem.summary <-summary(microbe.psem, .progressBar = F))

plot(microbe.psem)

######################## correlation matrix ###########################

jpeg("Fig.S1.jpeg", units="cm", width=24, height=20, quality=100, res=300)

d <- read_excel("plot0410.xls", sheet = "forest_cor")

cord <- round(cor(d), 2)

pd <-cor.mtest(d, conf.level= 0.95)

F2S1 <- ggcorrplot(cord, p.mat = cor_pmat(d), hc.order = FALSE, type = "lower",lab = TRUE,

lab_size = 4, pch.col="black", pch.cex = 4,

digits= 4, sig.level = 0.05,

#ggtheme = ggplot2::theme_gray,

ggtheme = ggplot2::theme_classic,

legend.title = "Pearson Correlation",

#colors = c("#6D9EC1", "white", "#E46726"),

outline.col = "white")

plot(F2S1)

plot_grid(F2S1, ncol=1)

dev.off()

jpeg("Fig.S2.jpeg", units="cm", width=24, height=20, quality=100, res=300)

d <- read_excel("plot0410.xls", sheet = "shrub_cor")

cord <- round(cor(d), 2)

pd <-cor.mtest(d, conf.level= 0.95)

F2S2 <- ggcorrplot(cord, p.mat = cor_pmat(d), hc.order = FALSE, type = "lower",lab = TRUE,

lab_size = 4, pch.col="black", pch.cex = 4,

digits= 4, sig.level = 0.05,

#ggtheme = ggplot2::theme_gray,

ggtheme = ggplot2::theme_classic,

legend.title = "Pearson Correlation",

#colors = c("#6D9EC1", "white", "#E46726"),

outline.col = "white")

plot(F2S2)

plot_grid(F2S2, ncol=1)

dev.off()

################## scatter plot for bivariate relationships

d1 <- read_excel("plot0410.xls", sheet = "scatter plot")

jpeg("Fig.3.jpeg", units="cm", width=60, height=40, pointsize=25, quality=100, res=300)

Fig.2a <- ggplot(d1, aes(x=AL, y=C, colour=Type),scale_shape_identity())+

geom_smooth(method = 'lm', se=T, size=1.5, linetype=1)+theme_classic()+

ggpubr::stat_cor(aes(color = Type), method='pearson',size=8, label.x = -1,label.y.npc =0.1, p.accuracy = 0.001)

#scale_colour_manual(values = c("#FF9999","#00CC33","#9999FF"))

Fig.2a <- Fig.2a + geom_point(size=5, alpha = 0.5)

#stat_smooth(method="lm", se=T, color="black", size=1.5, linetype=1)

Fig.2a <- Fig.2a+xlab(expression(paste(italic("ln "), "(Altitude, m)")))+ylab(expression(paste(italic("ln "), "(Carbon storage, Mg ", ha^-1,")")))

Fig.2a <- Fig.2a+theme(axis.line = element_line(colour = "black", size = 1.2, linetype = "solid"))+

theme(axis.ticks = element_line(colour = "black", size = 2))

Fig.2a <- Fig.2a+theme(axis.text = element_text(size=16, colour = "black"))+

theme(axis.title=element_text(size=25))+ theme(legend.position="none")

#annotate("text", size=6, x =-2.2, y = 1.8, label = "P< 0.05", parse = TRUE)

plot(Fig.2a)

Fig.2b <- ggplot(d1, aes(x=TEM, y=C, colour=Type),scale_shape_identity())+

geom_smooth(method = 'lm', se=T,size=1.5, linetype=1)+theme_classic()+

ggpubr::stat_cor(aes(color = Type), method='pearson',size=8, label.x = -2.8, label.y.npc =0.1 , p.accuracy = 0.001)

#scale_colour_manual(values = c("#FF9999","#00CC33","#9999FF"))

Fig.2b <- Fig.2b + geom_point(size=5, alpha = 0.5)

#stat_smooth(method="lm", se=T, color="black", size=1.5, linetype=1)

Fig.2b <- Fig.2b+xlab(expression(paste(italic("ln "), "(Mean annual temperature, ℃)")))+ylab(expression(paste(italic("ln "), "(Carbon storage, Mg ", ha^-1,")")))

Fig.2b <- Fig.2b+theme(axis.line = element_line(colour = "black", size = 1.5, linetype = "solid"))+

theme(axis.ticks = element_line(colour = "black", size = 2))

Fig.2b <- Fig.2b+theme(axis.text = element_text(size=16, colour = "black"))+

theme(axis.title=element_text(size=25))+ theme(legend.position="none")

#annotate("text", size=6, x =-1.8, y = 1.8, label = "P< 0.05", parse = TRUE)

plot(Fig.2b)

Fig.2c <- ggplot(d1, aes(x=SM, y=C, colour=Type),scale_shape_identity())+

geom_smooth(method = 'lm', se=T, size=1.5, linetype=1)+theme_classic()+

ggpubr::stat_cor(aes(color = Type), method='pearson',size=8, label.x = -3.8, label.y.npc =0.1, p.accuracy = 0.001)

#scale_colour_manual(values = c("#FF9999","#00CC33","#9999FF"))

Fig.2c <- Fig.2c + geom_point(size=5, alpha = 0.5)

#stat_smooth(method="lm", se=T, color="black", size=1.5, linetype=1)

Fig.2c <- Fig.2c+xlab(expression(paste(italic("ln "), "(Soil moisture, 0.001 ", m^3/m^3,")")))+ylab(expression(paste(italic("ln "), "(Carbon storage, Mg ", ha^-1,")")))

Fig.2c <- Fig.2c+theme(axis.line = element_line(colour = "black", size = 1.5, linetype = "solid"))+

theme(axis.ticks = element_line(colour = "black", size = 2))

Fig.2c <- Fig.2c+theme(axis.text = element_text(size=16, colour = "black"))+

theme(axis.title=element_text(size=25))+ theme(legend.position="none")

#annotate("text", size=6, x =-2.5, y = 1.8, label = "P< 0.05", parse = TRUE)

plot(Fig.2c)

Fig.2d <- ggplot(d1, aes(x=STRUC, y=C, colour=Type),scale_shape_identity())+

geom_smooth(method = 'lm', se=T,size=1.5, linetype=1)+theme_classic()+

ggpubr::stat_cor(aes(color = Type), method='pearson',size=8, label.x = 0, label.y.npc =0.1, p.accuracy = 0.001)

#scale_colour_manual(values = c("#FF9999","#00CC33","#9999FF"))

Fig.2d <- Fig.2d + geom_point(size=5, alpha = 0.5)

#stat_smooth(method="lm", se=T, color="black", size=1.5, linetype=1)

Fig.2d <- Fig.2d+xlab(expression(paste(italic("ln "), "(Structural diversity, %)")))+ylab(expression(paste(italic("ln "), "(Carbon storage, Mg ", ha^-1,")")))

Fig.2d <- Fig.2d+theme(axis.line = element_line(colour = "black", size = 1.5, linetype = "solid"))+

theme(axis.ticks = element_line(colour = "black", size = 2))

Fig.2d <- Fig.2d+theme(axis.text = element_text(size=16, colour = "black"))+

theme(axis.title=element_text(size=25))+ theme(legend.position="none")

#annotate("text", size=6, x =-3.2, y = 1.8, label = "P< 0.05", parse = TRUE)

plot(Fig.2d)

Fig.2e <- ggplot(d1, aes(x=SP, y=C, colour=Type),scale_shape_identity())+

geom_smooth(method = 'lm', se=T, size=1.5, linetype=1)+theme_classic()+

ggpubr::stat_cor(aes(color = Type), method='pearson',size=8, label.x = -1, label.y.npc =0.01, p.accuracy = 0.001)

#scale_colour_manual(values = c("#FF9999","#00CC33","#9999FF"))

Fig.2e <- Fig.2e + geom_point(size=5, alpha = 0.5)

#stat_smooth(method="lm", se=T, color="black", size=1.5, linetype=1)

Fig.2e <- Fig.2e+xlab(expression(paste(italic("ln "), "(Species diversity, %)")))+ylab(expression(paste(italic("ln "), "(Carbon storage, Mg ", ha^-1,")")))

Fig.2e <- Fig.2e+theme(axis.line = element_line(colour = "black", size = 1.5, linetype = "solid"))+

theme(axis.ticks = element_line(colour = "black", size = 2))

Fig.2e <- Fig.2e+theme(axis.text = element_text(size=16, colour = "black"))+

theme(axis.title=element_text(size=25))+ theme(legend.position="none")

#annotate("text", size=6, x =-3, y = 1.8, label = "P< 0.05", parse = TRUE)

plot(Fig.2e)

plot_grid(Fig.2a, Fig.2b, Fig.2c,Fig.2d, Fig.2e,

ncol=3,labels ="auto",label_size =30, hjust = -0.8, vjust = 1.5)

dev.off()

################################

d1 <- read_excel("plot0410.xls", sheet = "scatter plot")

jpeg("Fig.4.jpeg", units="cm", width=60, height=40, pointsize=25, quality=100, res=300)

Fig.2f <- ggplot(d1, aes(x=SP, y=STRUC, colour=Type),scale_shape_identity())+

geom_smooth(method = 'lm', se=T, size=1.5, linetype=1)+theme_classic()+

ggpubr::stat_cor(aes(color = Type), method='pearson',size=8, label.x = -3,label.y.npc = 0.1, p.accuracy = 0.001)

#scale_colour_manual(values = c("#FF9999","#00CC33","#9999FF"))

Fig.2f <- Fig.2f +geom_point(size=5,alpha = 0.5)

#stat_smooth(method="lm", se=T, color="black", size=1.5, linetype=1)

Fig.2f <- Fig.2f+xlab(expression(paste(italic("ln "), "(Species diversity, %)")))+ylab(expression(paste(italic("ln "), "(Structural diversity, %)")))

Fig.2f <- Fig.2f+theme(axis.line = element_line(colour = "black", size = 1.5, linetype = "solid"))+

theme(axis.ticks = element_line(colour = "black", size = 2))

Fig.2f <- Fig.2f+theme(axis.text = element_text(size=16, colour = "black"))+

theme(axis.title=element_text(size=25))+theme(legend.position="none")

#annotate("text", size=6, x =-3.5, y = 1.8, label = "P< 0.05", parse = TRUE)

plot(Fig.2f)

Fig.2g <- ggplot(d1, aes(x=TEM, y=STRUC, colour=Type),scale_shape_identity())+

geom_smooth(method = 'lm', se=T,size=1.5, linetype=1)+theme_classic()+

ggpubr::stat_cor(aes(color = Type), method='pearson',size=8, label.x = -3,label.y.npc = 0.1,p.accuracy = 0.001)

#scale_colour_manual(values = c("#FF9999","#9999FF","#00CC33"),

#limits = c("Deciduous Broadleaved Forest", "Evergreen Needleleaved Forest",

#"Deciduous Broadleaved Shrub"))

Fig.2g <- Fig.2g + geom_point(size=5, alpha = 0.5)

#stat_smooth(method="lm", se=T, color="black", size=1.5, linetype=1)

Fig.2g <- Fig.2g+xlab(expression(paste(italic("ln "), "(Mean annual temperature, ℃)")))+ylab(expression(paste(italic("ln "), "(Structural diversity %)")))

Fig.2g <- Fig.2g+theme(axis.line = element_line(colour = "black", size = 1.5, linetype = "solid"))+

theme(axis.ticks = element_line(colour = "black", size = 2))

Fig.2g <- Fig.2g+theme(axis.text = element_text(size=20, colour = "black"))+theme(axis.title=element_text(size=25))+

theme(legend.text= element_text(size=16, colour = "black"),legend.title= element_text(size=18, colour = "black"),

legend.position=c(1.6, 0.5))

#annotate("text", size=6, x =-1.5, y = 1.8, label = "P< 0.05", parse = TRUE)

plot(Fig.2g)

Fig.h <- ggplot(d1, aes(x=SM, y=STRUC, colour=Type),scale_shape_identity())+

geom_smooth(method = 'lm', se=T,size=1.5, linetype=1)+theme_classic()+

ggpubr::stat_cor(aes(color = Type), method='pearson',size=8, label.x = -3, label.y.npc = 0.1, p.accuracy = 0.001)

#scale_colour_manual(values = c("#FF9999","#00CC33","#9999FF"))

Fig.h <- Fig.h + geom_point(size=5, alpha = 0.5)

#stat_smooth(method="lm", se=T, color="black", size=1.5, linetype=1)

Fig.h <- Fig.h+xlab(expression(paste(italic("ln "), "(Soil moisture, 0.001 ", m^3/m^3,")")))+ylab(expression(paste(italic("ln "), "(Structural diversity, %)")))

Fig.h <- Fig.h+theme(axis.line = element_line(colour = "black", size = 1.5, linetype = "solid"))+

theme(axis.ticks = element_line(colour = "black", size = 2))

Fig.h <- Fig.h+theme(axis.text = element_text(size=16, colour = "black"))+theme(axis.title=element_text(size=25))+

theme(legend.position="none")

#annotate("text", size=6, x =-2.2, y = 1.8, label = "P< 0.05", parse = TRUE)

plot(Fig.h)

Fig.i <- ggplot(d1, aes(x=TEM, y=SP, colour=Type),scale_shape_identity())+

geom_smooth(method = 'lm', se=T, size=1.5, linetype=1)+theme_classic()+

ggpubr::stat_cor(aes(color = Type), method='pearson',size=8, label.x = -3,label.y.npc = 0.1, p.accuracy = 0.001)

#scale_colour_manual(values = c("#FF9999","#00CC33","#9999FF"))

Fig.i <- Fig.i + geom_point(size=5, alpha = 0.5)

#stat_smooth(method="lm", se=T, color="black", size=1.5, linetype=1)

Fig.i <- Fig.i+xlab(expression(paste(italic("ln "), "(Mean annual temperature, ℃)")))+ylab(expression(paste(italic("ln "), "(Species diversity %)")))

Fig.i <- Fig.i+theme(axis.line = element_line(colour = "black", size = 1.5, linetype = "solid"))+

theme(axis.ticks = element_line(colour = "black", size = 2))

Fig.i <- Fig.i+theme(axis.text = element_text(size=16, colour = "black"))+theme(axis.title=element_text(size=25))+

theme(legend.position="none")

#annotate("text", size=6, x =-2.2, y = 1.8, label = "P< 0.05", parse = TRUE)

plot(Fig.i)

Fig.j <- ggplot(d1, aes(x=TEM, y=SM, colour=Type),scale_shape_identity())+

geom_smooth(method = 'lm', se=T, size=1.5, linetype=1)+theme_classic()+

ggpubr::stat_cor(aes(color = Type), method='pearson',size=8, label.x = -3,label.y.npc = 0.1,p.accuracy = 0.001)

#scale_colour_manual(values = c("#FF9999","#00CC33","#9999FF"))

Fig.j <- Fig.j + geom_point(size=5, alpha = 0.5)

#stat_smooth(method="lm", se=T, color="black", size=1.5, linetype=1)

Fig.j <- Fig.j+xlab(expression(paste(italic("ln "), "(Mean annual temperature, ℃)")))+ylab(expression(paste(italic("ln "), "(Soil moisture, 0.001 ", m^3/m^3,")")))

Fig.j <- Fig.j+theme(axis.line = element_line(colour = "black", size = 1.5, linetype = "solid"))+

theme(axis.ticks = element_line(colour = "black", size = 2))

Fig.j <- Fig.j+theme(axis.text = element_text(size=16, colour = "black"))+theme(axis.title=element_text(size=25))+

theme(legend.text= element_text(size=26, colour = "black"),legend.title= element_text(size=28, colour = "black"),

legend.position=c(1.6, 0.5))

#annotate("text", size=6, x =-2.2, y = 1.8, label = "P< 0.05", parse = TRUE)

plot(Fig.j)

plot_grid(Fig.2f, Fig.2g,Fig.h, Fig.i, Fig.j,

ncol=3,labels = c('f', 'g', 'h', 'i', 'j'), label_size =30, hjust = -0.8, vjust = 1.5)

dev.off()

# References

Ali, A., Lin, S.-L., He, J.-K., Kong, F.-M., Yu, J.-H., and Jiang, H.-S. (2019). Climate and soils determine aboveground biomass indirectly via species diversity and stand structural complexity in tropical forests. *Forest Ecology and Management* 432**,** 823-831.

Ali, A., Yan, E.-R., Chen, H.Y.H., Chang, S.X., Zhao, Y.-T., Yang, X.-D., and Xu, M.-S. (2016). Stand structural diversity rather than species diversity enhances aboveground carbon storage in secondary subtropical forests in Eastern China. *Biogeosciences* 13**,** 4627-4635.

Aponte, C., Kasel, S., Nitschke, C.R., Tanase, M.A., Vickers, H., Parker, L., Fedrigo, M., Kohout, M., Ruiz‐Benito, P., Zavala, M.A., Bennett, L.T., and Hickler, T. (2020). Structural diversity underpins carbon storage in Australian temperate forests. *Global Ecology and Biogeography* 29**,** 789-802.

Chen, S., Wang, W., Xu, W., Wang, Y., Wan, H., Chen, D., Tang, Z., Tang, X., Zhou, G., Xie, Z., Zhou, D., Shangguan, Z., Huang, J., He, J.S., Wang, Y., Sheng, J., Tang, L., Li, X., Dong, M., Wu, Y., Wang, Q., Wang, Z., Wu, J., Chapin, F.S., 3rd, and Bai, Y. (2018). Plant diversity enhances productivity and soil carbon storage. *Proc Natl Acad Sci U S A* 115**,** 4027-4032.

Guo, Y., Schob, C., Ma, W., Mohammat, A., Liu, H., Yu, S., Jiang, Y., Schmid, B., and Tang, Z. (2019). Increasing water availability and facilitation weaken biodiversity-biomass relationships in shrublands. *Ecology* 100**,** e02624.

Kazempour Larsary, M., Pourbabaei, H., Sanaei, A., Salehi, A., Yousefpour, R., and Ali, A. (2021). Tree-size dimension inequality shapes aboveground carbon stock across temperate forest strata along environmental gradients. *Forest Ecology and Management* 496**,** 119482.

Poorter, L., Van Der Sande, M.T., Thompson, J., Arets, E.J.M.M., Alarcón, A., Álvarez-Sánchez, J., Ascarrunz, N., Balvanera, P., Barajas-Guzmán, G., Boit, A., Bongers, F., Carvalho, F.A., Casanoves, F., Cornejo-Tenorio, G., Costa, F.R.C., De Castilho, C.V., Duivenvoorden, J.F., Dutrieux, L.P., Enquist, B.J., Fernández-Méndez, F., Finegan, B., Gormley, L.H.L., Healey, J.R., Hoosbeek, M.R., Ibarra-Manríquez, G., Junqueira, A.B., Levis, C., Licona, J.C., Lisboa, L.S., Magnusson, W.E., Martínez-Ramos, M., Martínez-Yrizar, A., Martorano, L.G., Maskell, L.C., Mazzei, L., Meave, J.A., Mora, F., Muñoz, R., Nytch, C., Pansonato, M.P., Parr, T.W., Paz, H., Pérez-García, E.A., Rentería, L.Y., Rodríguez-Velazquez, J., Rozendaal, D.M.A., Ruschel, A.R., Sakschewski, B., Salgado-Negret, B., Schietti, J., Simões, M., Sinclair, F.L., Souza, P.F., Souza, F.C., Stropp, J., Ter Steege, H., Swenson, N.G., Thonicke, K., Toledo, M., Uriarte, M., Van Der Hout, P., Walker, P., Zamora, N., and Peña-Claros, M. (2015). Diversity enhances carbon storage in tropical forests. *Global Ecology and Biogeography* 24**,** 1314-1328.

Ren, S., Ali, A., Liu, H., Yuan, Z., Yang, Q., Shen, G., Zhou, S., and Wang, X. (2021). Response of community diversity and productivity to canopy gap disturbance in subtropical forests. *Forest Ecology and Management* 502**,** 119740.

Tetemke, B.A., Birhane, E., Rannestad, M.M., and Eid, T. (2021). Species diversity and stand structural diversity of woody plants predominantly determine aboveground carbon stock of a dry Afromontane forest in Northern Ethiopia. *Forest Ecology and Management* 500**,** 119634.

Ullah, F., Gilani, H., Sanaei, A., Hussain, K., and Ali, A. (2021). Stand structure determines aboveground biomass across temperate forest types and species mixture along a local-scale elevational gradient. *Forest Ecology and Management* 486**,** 118984.

Wen, Z., Jiang, Z., Zheng, H., and Ouyang, Z. (2022). Tropical forest strata shifts in plant structural diversity-aboveground carbon relationships along altitudinal gradients. *Sci Total Environ* 838**,** 155907.

Yan, G., Bongers, F.J., Trogisch, S., Li, Y., Chen, G., Yan, H., Deng, X., Ma, K., and Liu, X. (2022). Climate and mycorrhizae mediate the relationship of tree species diversity and carbon stocks in subtropical forests. *Journal of Ecology* 110**,** 2462-2474.

Yi, S., Wu, P., Peng, X., Tang, Z., Bai, F., Sun, X., Gao, Y., Qin, H., Yu, X., Wang, R., Du, N., and Guo, W. (2021). Biodiversity, environmental context and structural attributes as drivers of aboveground biomass in shrublands at the middle and lower reaches of the Yellow River basin. *Sci Total Environ* 774**,** 145198.

Yuan, Z., Ali, A., Sanaei, A., Ruiz-Benito, P., Jucker, T., Fang, L., Bai, E., Ye, J., Lin, F., Fang, S., Hao, Z., and Wang, X. (2021). Few large trees, rather than plant diversity and composition, drive the above-ground biomass stock and dynamics of temperate forests in northeast China. *Forest Ecology and Management* 481**,** 118698.
